# Catheter Ablation of Right-Sided Accessory Pathways in Adults Using the Three-Dimensional Mapping System: A Randomized Comparison to the Conventional Approach

**DOI:** 10.1371/journal.pone.0128760

**Published:** 2015-06-17

**Authors:** Yuedong Ma, Jia Qiu, Yang Yang, Anli Tang

**Affiliations:** 1 Department of Cardiology, the First Affiliated Hospital of Sun Yat-Sen University, Guangzhou, Guangdong, China; 2 Department of Pathology, the First Affiliated Hospital of Sun Yat-Sen University, Guangzhou, Guangdong, China; Sapienza University of Rome, ITALY

## Abstract

Three-dimensional (3D) mapping and navigation systems have been widely used for the ablation of atrial fibrillation and ventricular tachycardia, but the applicability of these systems for the ablation of supraventricular tachycardia (SVT) due to right-sided accessory pathways (RAPs) remains unknown. The goal of this prospective randomized study was to compare the safety, efficiency, and efficacy of nonfluoroscopic and conventional fluoroscopic mapping techniques in guiding catheter ablation of SVT due to RAPs. Of the 393 consecutive patients with SVT who were randomized to receive either conventional fluoroscopic or Ensite NavX mapping-guided ablation, 64 patients with RAPs were included for analysis. Endpoints for ablation were no evidence of RAP conduction and no inducible atrioventricular reentrant tachycardia (AVRT). The 3D group showed fewer ablation pulses and a shorter total ablation time compared to the conventional group, although the acute procedural success did not differ significantly between the two groups. Total procedure time, electrophysiological study time, total fluoroscopy time, and cumulative radiation doses were also significantly reduced in the 3D group. Patients in the conventional group with a right atrium diameter (RAD) ≥ 47 mm required a longer fluoroscopy time. There was no significant difference in the recurrence rates between the two groups over a follow-up period of 3 to 29 months. There were no permanent complications. The 3D mapping system may be a preferred alternative for patients with AVRT due to RAPs, especially for patients with a large RAD (≥ 47 mm).

## Introduction

Radiofrequency catheter ablation (RFCA) is an effective curative therapy for atrioventricular reentrant tachycardia (AVRT) supported by accessory pathways (APs). Reported success rates of left-sided AP ablation exceed 92%, and recurrence rates range from approximately 2% to 5%. In contrast, patients receiving right-sided accessory pathway (RAP) ablation have shown lower success rates of 67% to 100% and higher recurrence rates of approximately 9% to 16.7% [1].

The challenge of RAPs ablation lies in the unique anatomical characteristics such as the absence of a venous structure paralleling the tricuspid annulus (TA), greater circumference than the mitral valve, and the difference in angle with which the valve attaches to the TA [1]. These anatomical characteristics lead to difficulty in defining the ablation target and positioning catheters. In addition to low success rates, these properties lead to remarkably prolonged exposure to radiation for patients and laboratory staff, which could result in skin injury, radiation-induced cancer, and genetic malformations [2–4].

Ensite NavX is a three-dimensional (3D) electronic navigation system that has been successfully used to track the precise position of the tip of the mapping and ablation catheter tips. The system generates spatially accurate activation maps, enhancing the safety, efficiency, and efficacy of catheter ablation [5]. It has been widely used to ablate complicated arrhythmias, such as atrial fibrillation and ventricular tachycardia [2], and its safety and efficiency have been investigated in the ablation of uncomplicated arrhythmias, including atrioventricular nodal reentrant tachycardia (AVNRT) and AVRT [3, 4]. However, little is known about the utility of 3D mapping systems for specifically ablating AVRT due to RAPs. Although retrospective studies have suggested that NavX can reduce radiation exposure during supraventricular tachycardia (SVT) ablation in both pediatric and adult patients [1, 6], no direct, prospective, and randomized studies have compared 3D mapping system and conventional fluoroscopic mapping to investigate potential differences in the efficiency, efficacy, and safety of the two approaches for RAPs catheter ablation. Therefore, this small, prospective, and randomized study sought to compare the effectiveness of traditional fluoroscopic and 3D approaches for this clinical problem.

## Methods

The protocol for this trial and supporting CONSORT checklist are available as supporting information; see S1 CONSORT Checklist, S1 Protocol (English) and S2 Protocol (Chinese).

This study was conducted according to the Helsinki Declaration and approved by the Ethics Committee of Sun Yat-Sen University, Guangzhou, China (January 07, 2008) (S1 Ethical Approval Document). The study was registered with the Chinese Clinical Trial Registry (http://www.chictr.org, ChiCTR-TRC-08000268). The authors confirm that all ongoing and related trials for this intervention have been registered. Randomization was performed according to a computer-generated randomization scheme in blocks of four (S1 Protocol). Assignments were concealed in opaque, sealed envelopes that were numbered consecutively.

### Study population

The study included consecutive patients with documented paroxysmal SVT who were admitted to our institutions from June 2011 to November 2013. Thereafter, 393 patients who had no structural heart disease were randomized to receive either conventional fluoroscopic (conventional group) or Ensite NavX (3D group) mapping to guide catheter ablation, which was performed by a single, experienced operator using a standard procedure [5]. All antiarrhythmic drugs were discontinued for at least 5 half-lives before electrophysiological studies (EPS) were performed. Written informed consent for the procedure was provided by all patients. Patients with AVNRT, left-sided APs, atrial ectopic tachycardia, intra-atrial reentry tachycardia or junctional ectopic tachycardia were excluded. Finally, 64 patients with AVRT due to RAPs were analyzed in this study.

### Echocardiographic study

Transthoracic echocardiography was performed on an echocardiograph (Vivid 7, GE Healthcare, Milwaukee, MI, USA) equipped with a 2–4 MHz linear transducer with patients in the left lateral decubitus position before and after the RFCA procedure. The right atrium diameter (RAD) was measured at end systole in the apical four-chamber view. The rest of the procedure was performed through standard protocols and procedures, according to the guidelines of the American Society of Echocardiography [7]. A post-procedural echocardiogram was performed on all patients to exclude pericardial effusion or other acute complications.

### EP procedure

Each patient underwent the EP procedure while fasting and without sedation.

#### Conventional fluoroscopic mapping

Under local anesthesia, venous access was obtained from the femoral, right internal jugular, or subclavian veins to introduce diagnostic electrode catheters into the high right atrium, right ventricle, bundle of His, and coronary sinus. Ablation catheter access to the heart was achieved via the femoral vein. Fluoroscopy was used throughout all phases of the procedure, including confirmation of the guidewire position, EPS, mapping, and ablation.

#### 3D electronic navigation system

Seven skin patches were applied to guide the non-fluoroscopic Ensite NavX navigation system (version 8.0; St. Jude Medical, St Paul, MN, USA), as previously described [5]. The point clouds feature of the Ensite NavX system was used to position the diagnostic and ablation catheters through the right femoral vein and, if necessary, through the right internal jugular vein and/or left subclavian vein as in the fluoroscopic mapping procedure [5]. Briefly, the right atrium was reached and confirmed by the presence of atrial electrograms. The catheter was advanced and pulled back to mark the superior vena cava, followed by the inferior vena cava. After the right ventricle was reached, the catheter was pulled back with clockwise rotation to the right atrioventricular annulus (A and V wave amplitudes at roughly equal levels) and then delivered to the coronary sinus. Other diagnostic (high right atrium, right ventricle, and His bundle) or ablation catheters were placed with the point clouds technique. Thus, a rough sketch of the right atrial geometry was constructed from the placed catheters. The coronary sinus catheter served as a positional reference for the remainder of the procedure. An adequate, 3D image of the right atrium was completed by moving the ablation catheter for several minutes. Technical details of the electroanatomical mapping system (NavX) have been previously described [1, 5–7].

The EP study and ablation were performed in accordance with standard protocols and procedures [5]. Briefly, during sinus rhythm or atrial pacing, NavX showed a breakout of activation around the tricuspid annulus simultaneously with or just before onset of the delta wave. Radiofrequency energy was applied at the breakouts, with power and temperature limits of 40 W and 55°C, respectively. Radiofrequency energy was applied via a 7-F, 4-mm electrode-tipped steerable ablation catheter (Stinger, Bard EP, Lowell, MA, USA). The endpoint of the procedure was persistent absence of both retrograde and antegrade pathway conduction. When necessary for orienting or confirming the catheter location, fluoroscopy was performed with a radiographic/fluoroscopic unit (Innova 2100, GE Healthcare, Waukesha, WI, USA). The minimum fluoroscopy dose compatible with adequate imaging was used.

### Procedure and fluoroscopy times

The *preparation time* was calculated from the time the patient entered the procedure room until the time of the beginning of needle puncture. The *geometry time* was measured from the insertion of the first catheter until the beginning of the EP study. The *EP study time* was defined as the interval from the beginning of initial premature extrastimuli until the definition of the ablation target. The *total procedure time* was defined as the interval from the placement of the first venous sheath until the removal of the last sheath from the patient. The *numbers of ablation pulses* and *total ablation time* (i.e., total time that ablation energy was on) were recorded. The *total fluoroscopy time* was defined as the cumulative duration of fluoroscopy during the entire procedure. The *cumulative radiation dose* was calculated as the total dose received by the patient.

### Acute success and procedural complications

Acute success was defined as follows: 1) no evidence of RAP conduction, and 2) no related AVRT could be induced for more than 30 minutes after the last radiofrequency energy application, under basal conditions or with intravenous isoprenaline, accompanied by documentation of transient atrioventricular block with adenosine. Complications were defined as permanent second- or third-degree atrioventricular block, vascular or cardiac injury, and pericardial effusion.

### Postablation assessment and follow-up

A 12-lead electrocardiogram (ECG) was performed for all patients when the patient returned to the ward from the catheter room. Patients received a physical examination and ECG prior to discharge. Arrhythmia recurrence was documented by ECG and patient diary records. In detail, patients were assessed at 1, 6, 12, 18, 24, and 30 months after RFCA by clinical evaluation with standard ECG and ECG Holter monitoring. An exercise stress test was performed on consenting patients. Telephone contact was maintained with patients throughout the entire study to assess the long-term recurrences of symptoms. Recurrence was defined as a relapse of pre-excitation (delta-waves) due to RAPs, ECG-documented tachycardia, or return of clinical symptoms that were identical to those before ablation and eventually proven to be AVRT due to RAPs by the subsequent EP study.

### Statistical analyses

Data are reported as means ± standard deviations (SDs). Continuous variables were compared by using an independent sample *t*-test. Pearson’s chi-square (χ^2^) and Fisher’s exact tests were used as appropriate to compare categorical values. Correlations between variables were assessed with Pearson’s correlation coefficient. Receiver operator characteristic (ROC) curves were constructed for RAD as a predictor of total fluoroscopy time, and the area under the ROC curve (AUC) was calculated. The Youden index was used to calculate optimal cutoff points for RAD. A *P*-value < 0.05 was considered statistically significant. Statistical analyses were performed with SPSS 10.0 software (SPSS Inc., Chicago, IL, USA).

## Results

### Patient characteristics

**Fig 1:**
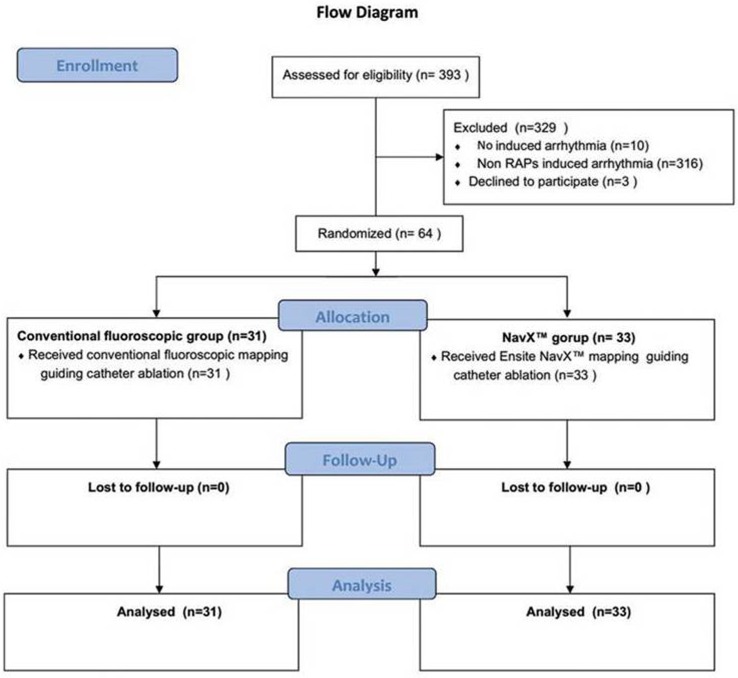
Flow diagram.

A total of 393 ablation procedures were performed during the study period (Fig 1). In total, 64 patients (40 males, and 24 females; age range: 18 to 52 years; mean age: 36.1 ± 8.1 years) with RAPs met the inclusion criteria and were analyzed in this study, including 31 patients in the conventional group and 33 patients in the 3D group. Distributions of gender, age, weight, left ventricular ejection fraction, RAD, underlying heart disease, substrate location, AVRT type, and AP location were not significantly different between the two groups (Table 1).

**Table 1:** Baseline and electrophysiological characteristics for patients in the conventional and 3D groups.

Baseline characteristics	Conventional	3D	P
Number of patients	31	33	N/A
Male/Female	19/12	21/12	0.846
Age (years)	37.7±8.7	33.6±7.3	0.127
Weight (kg)	59.5±7.6	60.1±7.8	0.778
Left ventricular ejection fraction (%)	74.5±5.1	72.9±6.2	0.259
Right atrium diameter (mm)	44±6	47±7	0.114
Underlying heart disease			
Congenital heart disease	0	0	
Mitral valve prolapse	0	1 (mild)	
Coronary artery disease (n)	0	0	
Hypertension (n)	2	3	0.9999
Type of AVRT			
Manifest accessory pathways (n)	22	22	0.711
Concealed accessory pathways (n)	9	11	
Location of accessory pathways			
Anterior septum (n)	5	5	0.9999
Middle septum (n)	5	8	0.529
Posterior septum (n)	8	7	0.714
Right ventricular free wall (n)	13	13	0.836

Note: AVRT, atrioventricular reentrant tachycardia; The tricuspid annulus is oriented as a clock face, where the anterior septum is located from 12:00 to 2:00, middle septum from 2:00 to 5:00, posterior septum from 5:00 to 7:00, and right ventricular free wall from 7:00 to 12:00.

### Procedural data

Outcomes from the EP procedure are shown in Table 2. There was no significant difference in the acute success rate of ablation between the 3D group and the conventional group (100% vs. 90.3%, *P* > 0.05). Ablation failed in 3 patients with anterior or middle septal RAPs, due to transient third-degree atrioventricular block. One patient in the conventional group experienced recurrence 2 days after the procedure. Two patients in the 3D group had recurrences 15 and 45 days after the procedure, respectively. Cases that failed or recurred were then successfully ablated under NavX guidance. The preparation and geometry times were not significantly different between the groups. However, the EP study and total procedure times were significantly shorter in the 3D group. The 3D group exhibited significantly less ablation-related damage to the heart, as indicated by fewer ablation pulses and a shorter total ablation time than the conventional group.

**Table 2:** Comparison of procedural variables between the conventional and 3D groups.

Procedural variables	Conventional	3D	P
Acute success rate, n (%)	28/31 (90.3%)	33/33 (100%)	0.106
Recurrence, n (%)	1/28 (3.6%)	2/33 (6.1%)	0.9999
Preparation time	13.5±1.3	14.2±1.9	0.089
Geometry time	21.3±3.7	23.1±4.0	0.057
EP study time	45.7±8.8	37±5.1	<0.001
Total procedure time	137.2±12.3	119.3±14.1	<0.001
Total ablation time	4.2±1.5	2.3±0.7	<0.001
Numbers of ablation pulses (n)	10.9±4.3	4.4±2.4	<0.001
Total fluoroscopy time (minutes)	22.9±6.2	4.0±3.7	<0.001
Cumulative radiation doses (mGy)	53.8±7.6	8.8±6.7	<0.001

Note: EP, electrophysiological

**Fig 2:**
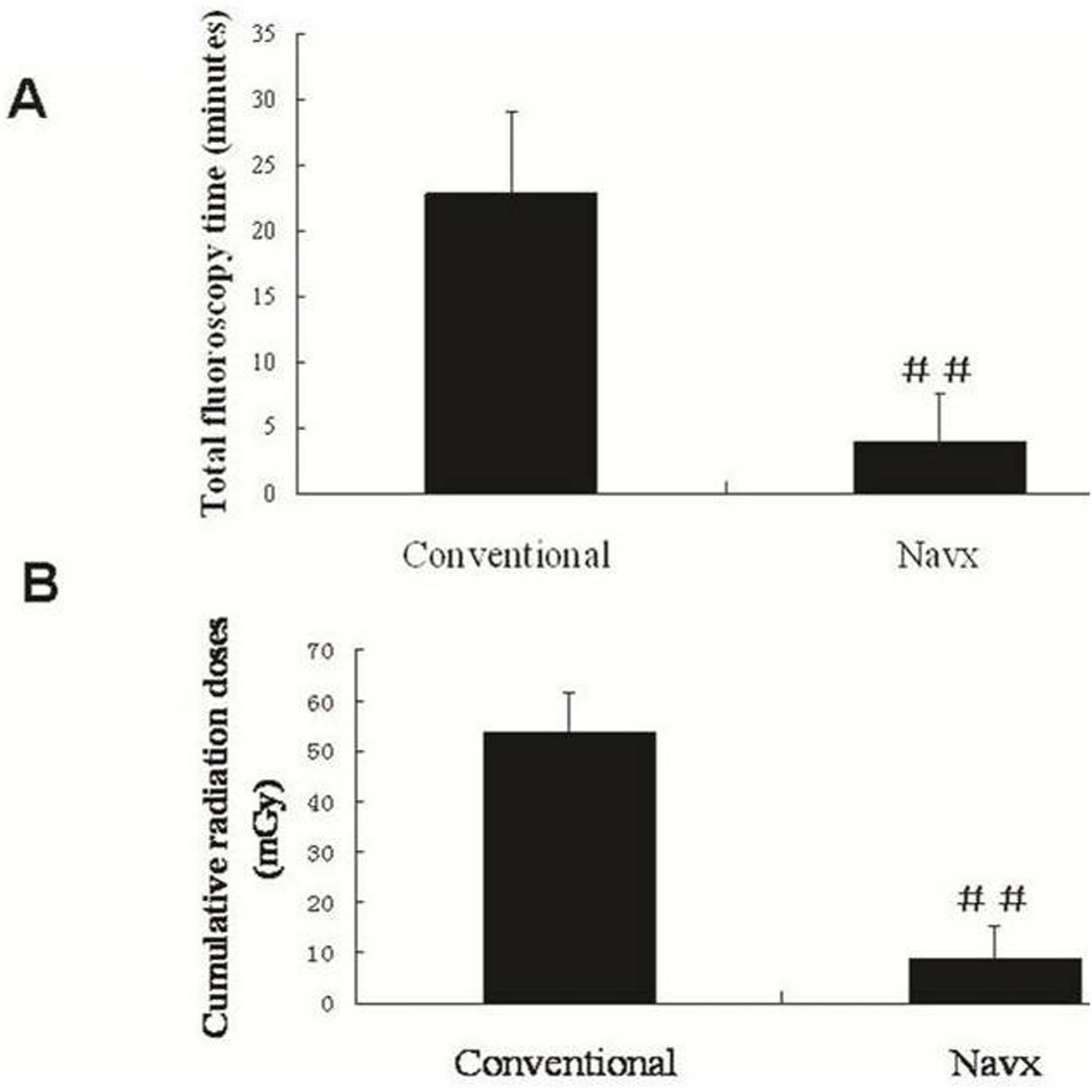
Comparison of fluoroscopy exposure between the conventional and 3D (NavX) groups. ^##^ *p<*0.001

**Fig 3:**
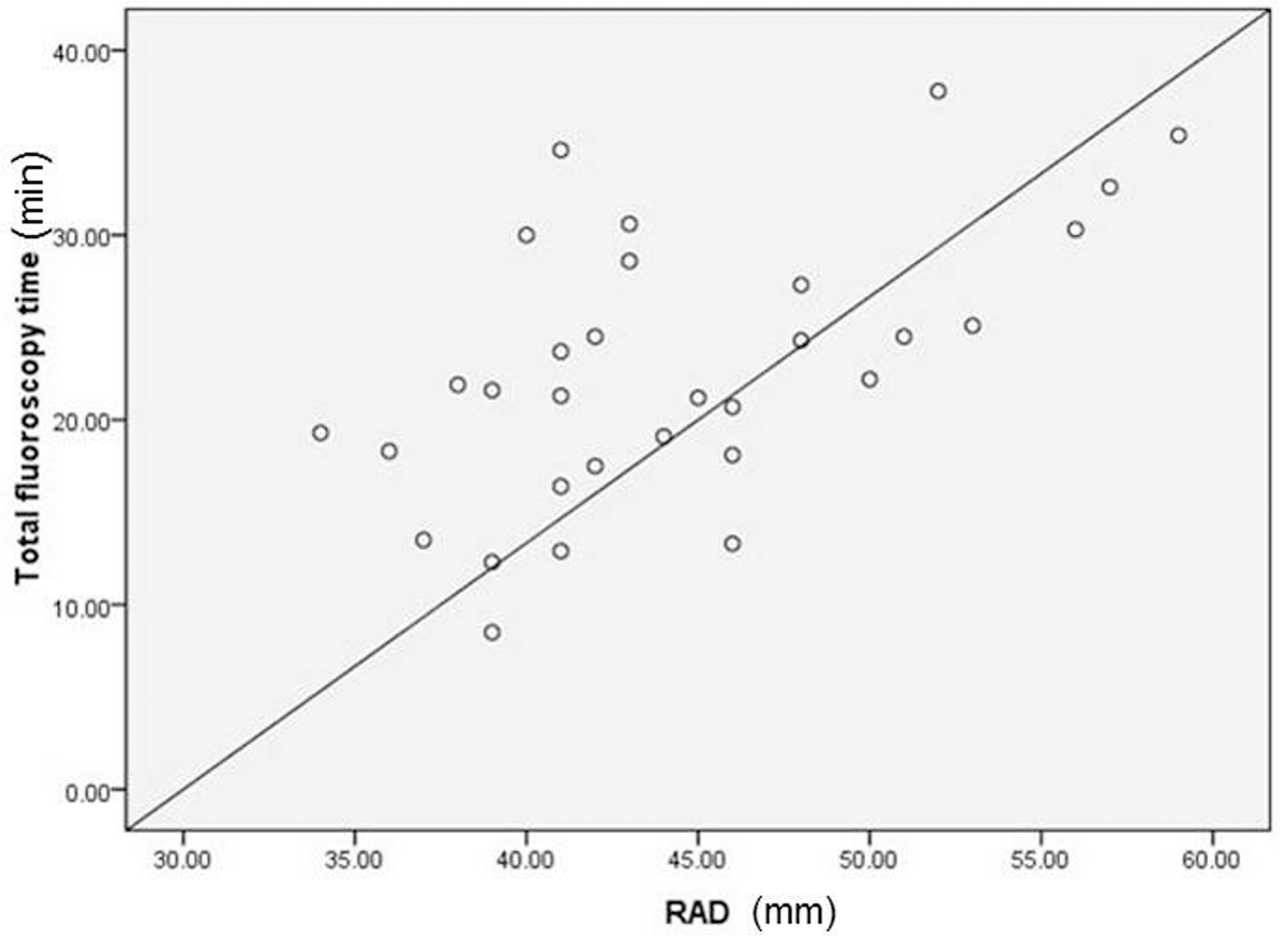
Positive linear correlation between RAD and total fluoroscopy time in the conventional group. R = 0.575, P = 0.01.

**Fig 4:**
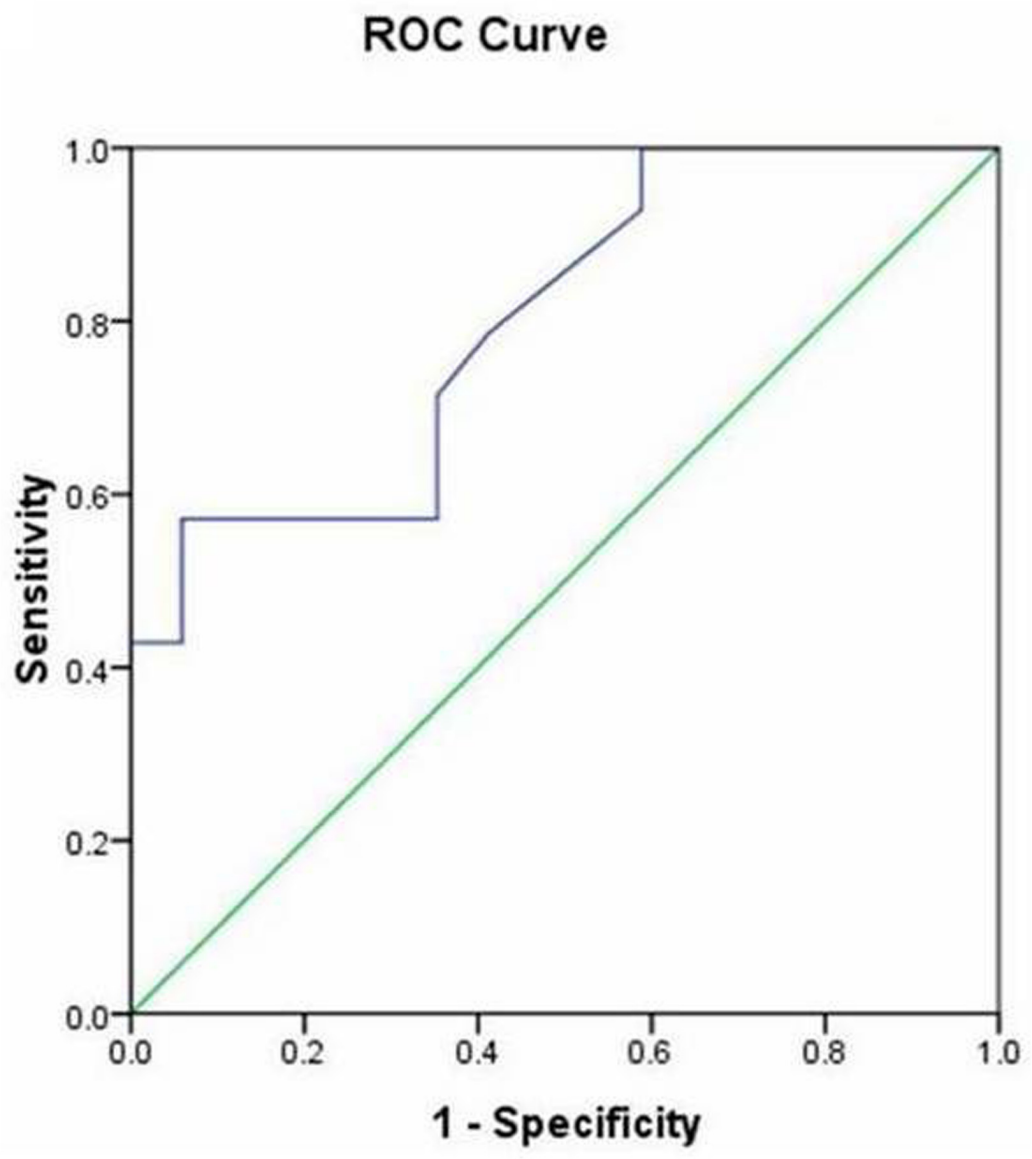
ROC curve (data from the conventional group). AUC = 0.8, P = 0.005, cut-off point = 47 mm.

Compared to the conventional group, the 3D group showed reductions in the total fluoroscopy time (4 ± 3.7 min vs. 22.9 min ± 6.2, *P* < 0.001) and the cumulative radiation doses (8.8±6.7 vs. 53.8±7.6 mGy, *P* < 0.001; Fig 2). Correlation analysis showed a positive linear correlation between RAD and the total fluoroscopy time in the conventional group (r = 0.575, *P* = 0.01; Fig 3), but not in the 3D group (r = 0.318, *P* = 0.71). The distributions of gender, age, weight, and left ventricular ejection fraction were not correlated with total fluoroscopy time in the conventional group. RAD was a good predictor of the total fluoroscopy time, according to the AUC [0.8; 95% confidence interval (0.645 0.955), *P* = 0.005; Fig 4]. The cut-off point of RAD was 47mm, according to Youden index. There were 9 and 16 patients with RAD ≥ 47 mm in the conventional group and the 3D group, respectively. There were no significant complications in either group.

## Discussions

The current study demonstrates the advantage of using the 3D non-fluoroscopic mapping system to guide RAP ablation in adults. There were no differences in the preparation or geometry time between the two approaches; however, use of the 3D system significantly reduced the total procedure time, EP study, and fluoroscopy times, as well as the cumulative radiation doses, especially in cases with a large RAD (≥ 47 mm). To the best of our knowledge, this is the first randomized and prospective report of the use of a 3D system for RAP ablation in a non-pediatric population.

RFCA is an established treatment for tachyarrhythmia [5]. Conventionally, catheter manipulation has been performed under a fluoroscopically guided approach, especially for paroxysmal SVT. The catheter ablation procedure is often complex and sufficiently prolonged to involve a non-negligible radiation exposure [8, 9]. Given the relationship between the radiation dose from medical imaging and the attributable lifetime risk of cancer and genetic anomalies [8], it is important to minimize X-ray exposure in cardiac EP practice. Ensite NavX was recently developed [10] as a nonfluoroscopic 3D mapping system to guide advanced EP procedures. However, owing to its time-consuming and expensive nature, the system has been used infrequently for less complex ablation procedures, such as the ablation of AVRT or AVNRT. In the present study, use of this 3D mapping system for RAP ablation significantly reduced the total procedure and EP study times compared to the conventional procedure, although there was an increasing trend in preparation time. Reductions in the total procedure and EP study times were mainly due to faster and more accurate determination of the ablation target, and easier positioning of the ablation catheter, especially in patients with a large RAD (≥47mm). However, the 3D group did not show a clear trend of an increased acute success rate of ablation. Two factors that may have contributed to this result are as follows. First, patients enrolled in this study did not have any associated structural heart disease. Thus, the differences could be even greater if patients with structural heart disease had been included. Second, the operator in this study was an electrophysiology expert with over 30 years of experience in RFCA of paroxysmal SVT. Because of transient atrioventricular block, two cases with anterior septal AP and one with middle septal AP failed in the conventional group, due to the close proximity of the AP and the bundle of His.

RAPs are thought to be distributed mainly in the free wall and posterior septum, whereas APs located in anterior and middle septal are rare [11]. In our study, anterior septal RAPs, near the bundle of His, were fairly common. The 3D system had more advantages for these patients with para-His RAPs, since the three failed cases with anterior or middle septal RAPs were then successfully treated guided by 3D system with no subsequent recurrence and complication.

Interestingly, we found that the RAD had a linear correlation with the total fluoroscopy time in the conventional group. The RAD can also be used as a basis for choosing the operation approach, because it was a good predictor of fluoroscopy time according to the ROC analysis and had an optional cutoff point of 47 mm.

Statistical limitations make it difficult to evaluate the relationship between long-term cancer risk and low doses of radiation, but the Biological Effects of Ionizing Radiation VII (BEIR VII) of the US National Academies concluded that current evidence supports a “linear-no-threshold” model. According to this model, a simple linear relationship exists between cancer risk and radiation dose [5, 10], and there is no threshold dose below which radiation carries no risk. Even low doses of radiation may have appreciable noxious effects, as evidenced by the observation of both acute and long-term DNA damage in circulating lymphocytes of children undergoing cardiac catheterization [12]. It also bears particular importance to note that radiation risks are not distributed homogeneously among the population, as women and younger individuals are at relatively higher risk, due to their greater vulnerability to radiation effects and longer life expectancy [10, 13, and 14]. Therefore, the greatest benefits of applying the 3D system in RAP ablation are the significant reduction in fluoroscopy time and radiation dose (Fig 2).

In addition to reduced radiation exposure, 3D system-guided RAP ablation has other advantages over the conventional procedure. It allows for elucidation of detailed individual variations in anatomy and electrogram distributions, and has the ability to “tag” important sites, such as catheter locations and lesion sites, which can be revisited with great accuracy. The system can create “shadows” of the catheters, which can be used for the repositioning of the catheters in the case of dislodgment. Thus, compared to the conventional fluoroscopy-guided procedure, 3D system can be used to ablate RAPs more effectively, with fewer radiofrequency applications, lower radiofrequency energy, shorter procedure time, and reduced risk of inadvertent atrioventricular block.

Although 3D mapping costs more than conventional mapping due to the electrodes used, the extra cost is balanced by the added benefits, such as reduction in total procedure time and radiation exposure. Moreover, the cost may go down in the future if electrodes can be recycled.

### Study Limitations

This study involved a relatively small number of subjects. Therefore, the low complication rate can not be statistically evaluated. A much larger sample size will be necessary to determine whether or not this approach will have any impact on the complication rates. The follow-up period was also short in some cases (range: 3–29 months). However, the follow-up period was long enough to judge the outcome of ablation because most recurrences reportedly occur within 3 months after ablation [15]. In the present study, all recurrences in both groups occurred within the first 2 months after ablation.

## Conclusions

This prospective, randomized, single-center study demonstrated that the 3D mapping approach significantly reduced fluoroscopy exposure, although it did not show a significant trend of an increased acute success rate of RAP ablation in an adult population. Cases with anterior or middle septal RAPs are not rare. The 3D mapping system may be more beneficial for these patients, especially those with a large RAD (≥47 mm).

## Supporting Information

S1 CONSORT Checklist (DOC)

S1 Ethical Approval Document (PDF)

S1 Protocol Study protocol in English. (DOCX)

S2 Protocol Study protocol in Chinese. (DOC)
