# Supplementary material for: Catheter Ablation of Right-Sided Accessory Pathways in Adults Using the Three-Dimensional Mapping System: A Randomized Comparison to the Conventional Approach
Source: PLoS One. 2015 Jun 17;10(6):e0128760. doi: 10.1371/journal.pone.0128760 (PMC4471277; doi:10.1371/journal.pone.0128760)
Supplement: S1 Ethical Approval Document — (PDF) [file pone.0128760.s002.pdf]

中山大学附属第一医院医学伦理委员会批复件

伦审技[2008]11号　　　　　　　　　　审议日期：2008年1月7日

审议项目	三维标测技术指导心房颤动的个体化射频消融。		
负责科室	心内科	项目负责人	唐安丽教授

出席委员	性别	单　　位	专业	签名
谢灿茂	男	中山大学附属第一医院呼吸内科	临床医学、管理学	
刘全樑	男	中山大学附属第一医院医务处	医院管理学	
黎曙霞	女	中山大学附属第一医院药学部	临床药学	
万洪富	男	广东省生态环境与土壤研究所	环境与土壤/省人大常委	
刘秋生	男	中山大学附属第一医院医务科	医院管理学	
许　庚	男	中山大学附属第一医院耳鼻喉科	临床医学	
祁少海	男	中山大学附属第一医院设备科	医院管理学	
任　焰	女	中山大学政务学院社会学系	社会学	
成守珍	女	中山大学附属第一医院护理部	护理学	
陈旻湖	男	中山大学附属第一医院消化内科	临床医学	
何裕隆	男	中山大学附属第一医院胃肠外科	临床医学	
张武军	男	中山大学附属第一医院院区医务办	医院管理	
章海山	男	中山大学伦理学与哲学教研室	伦理学、哲学	
蔡海宁	男	广东经纶律师事务所	法律、律师	
何晓顺	男	中山大学附属第一医院肝移植科	临床医学	
杨建勇	男	中山大学附属第一医院放射科	医学影像	
骆福添	男	中山大学公共卫生学院统计学教研室	卫生统计学	

已提交审阅的材料	1、中山大学临床医学研究 5010 计划课题建议书。<br>2、中山大学临床医学研究 5010 计划项目任务书。<br>3、临床医学研究知情同意书。<br>4、中山一院伦理评审受理表。
投票结果	1.同意（16）人　2.作必要的修改后同意（1）人；<br>3.不同意（0）人 4.终止或暂停先前已批准的试验（0）人。

医学伦理委员会意见：

1、经会议审议，心内科唐安丽教授负责的三维标测技术指导心房颤动的个体化射频消融研究，符合医学伦理原则及道德要求，原则同意研究者按本研究方案及知情同意书开展临床研究。

2、建议知情同意书使用通俗语言。

3、在项目研究过程中，应遵守国际《赫尔辛基宣言》及我国卫生部制定的《涉及人的生物医学研究伦理审查办法（试行）》及相关伦理原则、道德标准与相关的法律、法规、常规、制度等。

4、如对研究方案或知情同意书进行修改应及时向医学伦理委员会书面报告，所涉及的伦理问题均应提交医学伦理委员会再审议。

主任委员签名：谢灿茂

批复日期：2008 年 1 月 7 日

中山大学附属第一医院 医学伦理委员会

声明：本委员会仅对审查与备案的临床研究项目中涉及的伦理与道德问题负责。

联系地址：广州市中山二路 58 号，中山大学附属第一医院医学伦理委员会，邮编：510080
联系电话：020-87775064、020-87755766 转 8037、8040；传　　真：020-87334602

中山大學 附属第一医院
THE FIRST AFFILIATED HOSPITAL SUN YAT-SEN UNIVERSITY

医学伦理委员会项目评审受理表

受理号：

项目名称	三维标测技术指导心房颤动的个体化射频消融				
项目负责人	唐安丽	所在科室	心内科	联系电话	13728015092
研究类别	□药物临床试验 □医疗器械临床验证 □人类辅助生殖技术<br>☑医疗新技术 □动物实验 ☑临床观察研究 □其它				
经费来源	中山大学临床医学研究“5010”计划项目				
研究目的与意义	心房颤动（房颤）是临床上常见的快速心律失常，是脑卒中、心力衰竭等致死、致残的重要原因。目前经导管射频消融治疗房颤已成为房颤治疗的重要手段。本项研究的目的是评价不同患者根据其房颤发生的不同机制采用个体化的消融的疗效及安全性。				
申报资料清单	□国家食品药品监督管理局（SFDA）或其它上级主管部门批件<br>□药品注册证 ☑临床试验方案 ☑受试者知情同意书 □研究者手册<br>□病历报告表 □质量检验报告 ☑研究者名单及简历 ☑研究合作协议书<br>□中心伦理委员会审批件 □试验产品已有的有效性和安全性资料 □产品使用说明书 □招募广告 □企业法人营业执照<br>□GMP 证 □产品生产许可证 □其他				
科室意见	同意。 [illegible]				
项目主管部门意见（此栏由药物临床研究基地或科研等主管部门填写）	同意报送伦理委员会审批！<br>中山大学附属第一医院 科研科<br>2007 年 12 月 28 日				
伦理委员会意见	□ 资料齐全，同意受理。<br>□ 资料不齐全，补充后受理。<br>□ 其他：				

2007 年 4 月 13 日制表
