# Supplementary material for: Catheter Ablation of Right-Sided Accessory Pathways in Adults Using the Three-Dimensional Mapping System: A Randomized Comparison to the Conventional Approach
Source: PLoS One. 2015 Jun 17;10(6):e0128760. doi: 10.1371/journal.pone.0128760 (PMC4471277; doi:10.1371/journal.pone.0128760)
Supplement: S1 Protocol — (DOCX) [file pone.0128760.s003.docx]

Study protocol

1. Trial details

1.1 Trial details

| Protocol Title | Catheter ablation of right-sided accessory pathways in adults using the three-dimensional mapping system |
| --- | --- |
| Trial Start Date | June 2011 |
| Trial Finish Date | November 2013 |
| Sample size | 60 |
| Study type | Interventional |
| Study design | Randomized parallel control |
| Primary sponsor | The First Affiliated Hospital of Sun Yat-Sen University |
| Primary sponsor's address | No. 58 Zhongshan Road Ⅱ, Guangzhou, Guangdong 510080, China |
| Study leader | Anli Tang |

1.2 Trial Summary

Three-dimensional (3D) mapping and navigation system has been widely used for the ablation of atrial fibrillation and ventricular tachycardia, but its applicability for the ablation of supraventricular tachycardia (SVT) due to right-sided accessory pathways (RAPs) remains unknown. The goal of this study will compare the safety, efficiency, and efficacy of nonﬂuoroscopic and conventional ﬂuoroscopic mapping in guiding catheter ablation of SVT due to RAPs. This is a single-center, parallel-design randomized controlled trial (RCT). Consecutive patients with SVT will be randomized to receive either conventional ﬂuoroscopic or Ensite NavX™ mapping guiding ablation during the period of June 2011 to November 2013, and patients with RAPs will be included for analysis. Endpoints for ablation are no evidence of RAPs conduction and no inducible atrioventricular reentrant tachycardia (AVRT). The primary outcome measures will be the fluoroscopy times and procedural success. The secondary outcomes will include procedure times, complications, the number of ablation pulses and total ablation time.

1. Background

Radiofrequency catheter ablation (RFCA) is an effective curative therapy for atrioventricular reentrant tachycardia (AVRT) supported by accessory pathways (APs). Reported success rates of left-sided AP ablation exceed 92%, and recurrence rates range approximately 2% to 5%. In contrast, patients receiving right-sided accessory pathway (RAP) ablation have shown lower success rates of 67% to 100% and higher recurrence rates of approximately 9% to 16.7% [1].

The challenge of RAPs ablation lies in the unique anatomical characteristics such as the absence of a venous structure paralleling the tricuspid annulus (TA), greater circumference than the mitral valve, and the difference in angle with which the valve attaches to the TA [1]. These anatomical characteristics lead to difficulty in defining the ablation target and positioning catheters. In addition to low success rates, these properties lead to remarkably prolonged exposure to radiation for patients and laboratory staff, which could result in skin injury, radiation-induced cancer, and genetic malformations [2-4].

Ensite NavX™ is a 3D electronic navigation system that has been successfully used to track the precise position of the tip of the mapping and ablation catheter tips. The system generates spatially accurate activation maps, enhancing the safety, efficiency, and efficacy of catheter ablation [5]. It has been widely used to ablate complicated arrhythmias, such as atrial fibrillation and ventricular tachycardia [2], and its safety and efficiency have been investigated in the ablation of uncomplicated arrhythmias, including atrioventricular nodal reentrant tachycardia (AVNRT) and AVRT [3, 4]. However, little is known about the utility of 3D mapping system for specifically ablating AVRT due to RAPs. Although retrospective studies have suggested that NavX™ can reduce radiation exposure during supraventricular tachycardia (SVT) ablation in both pediatric and adult patients [1, 6], no direct, prospective, and randomized studies have compared 3D mapping system and conventional ﬂuoroscopic mapping to investigate potential differences in the efficiency, efficacy, and safety of the two approaches for RAP catheter ablation.

1. Objectives

This small, prospective, and randomized study will compare the effectiveness of traditional ﬂuoroscopic and 3D approaches for this clinical problem.

1. Trial design

This is a randomized, single-center, statistician-blinded, controlled trial with two parallel arms. The trial will be conducted in the Department of Cardiology, The First Affiliated Hospital of Sun Yat-Sen University (a grade III level A teaching hospital), Guangzhou, Guangdong, China, between June 2011 to November 2013.This protocol has been approved by the ethics review boards of the First Affiliated Hospital of Sun Yat-Sen University. Written informed consent will be obtained from all study participants prior to enrolment.

Eligible patients will be randomized to the conventional ﬂuoroscopic and 3D group (Ensite NavX™ mapping

system guiding catheter ablation) (Fig. 1). Patients will be assessed at 1, 6, 12, 18, 24, and 30 months after RFCA by clinical evaluation with standard ECG and ECG Holter monitoring (Table 4). Results will be analyzed by professionals blinded to the group allocation. This protocol has been registered with the Chinese Clinical Trials Register, a registry in the WHO Registry Network.

4.1 Eligibility

**Inclusion criteria**

Participants will be included if they fulfil the following criteria: (1) over 18years old; (2) paroxysmal SVT due to RAP; (3) all antiarrhythmic drugs have been discontinued for at least five half-lives before electrophysiological studies (EPS) and (4) Written informed consent by patients.

**Exclusion criteria**

Patients with any of the following conditions will be excluded: (1) AVNRT, AVRT due to left-sided APs, atrial ectopic tachycardia, intra-atrial reentry tachycardia or junctional ectopic tachycardia; (2) structural heart diseases; (3) other severe diseases, such as pancreatitis, stroke, aortic dissection, liver or kidney diseases, and relevant serious complications.

4.2 Randomization and allocation concealment

In this trial, randomization will be performed according to a computer-generated randomization scheme, and with a block size of 4. Assignments will be concealed in opaque, sealed envelopes that were numbered consecutively. Assignment concealment will be broken only after the participant has met all selection criteria and completed the baseline assessments. The operator and participants will know the allocated group, but the researcher responsible for collecting and analyzing the outcomes will be blind to the grouping of subjects and will not participate in the operation treatment.

4.3 Intervention

**EP procedure**

Each patient will undergo the EP procedure while fasting and without sedation. Conventional ﬂuoroscopic mapping. Under local anesthesia, venous access will be obtained from the femoral, right internal jugular, or subclavian veins to introduce diagnostic electrode catheters into the high right atrium, right ventricle, bundle of His, and coronary sinus. Ablation catheter access to the heart will be achieved via the femoral vein. Fluoroscopy will be used throughout all phases of the procedure, including confirmation of the guidewire position, EPS, mapping, and ablation.

**3D electronic navigation system.** Seven skin patches will be applied to guide the non-fluoroscopic Ensite NavX™ navigation system (version 8.0; St. Jude Medical, St Paul, MN, USA), as previously described [5]. The point clouds feature of the Ensite NavX™ system will be used to position the diagnostic and ablation catheters through the right femoral vein and, if necessary, through the right internal jugular vein and/or left subclavian vein as in the ﬂuoroscopic mapping procedure [5]. Brieﬂy, the right atrium will be reached and conﬁrmed by the presence of atrial electrograms. The catheter will be advanced and pulled back to mark the superior vena cava, followed by the inferior vena cava. After the right ventricle has been reached, the catheter will be pulled back with clockwise rotation to the right atrioventricular annulus (A and V wave amplitudes at roughly equal levels) and then delivered to the coronary sinus. Other diagnostic (high right atrium, right ventricle, and His bundle) or ablation catheters will be placed with the point clouds technique. Thus, a rough sketch of the right atrial geometry will be constructed from the placed catheters. The coronary sinus catheter will serve as a positional reference for the remainder of the procedure. An adequate, 3D image of the right atrium will be completed by moving the ablation catheter for several minutes. Technical details of the electroanatomical mapping system (NavX™) have been previously described [1, 5-7].

The EP study and ablation will be performed in accordance with standard protocols and procedures [5]. Briefly, during sinus rhythm or atrial pacing, NavX™ will show a breakout of activation around the tricuspid annulus simultaneously with or just before onset of the delta wave. Radiofrequency energy will be applied at the breakouts, with power and temperature limits of 40 W and 55 ℃, respectively. Radiofrequency energy will be applied via a 7-F, 4-mm electrode-tipped steerable ablation catheter (Stinger, Bard EP, Lowell, MA, USA). The endpoint of the procedure is persistent absence of both retrograde and antegrade pathway conduction. When necessary for orienting or confirming the catheter location, fluoroscopy will be performed with a radiographic/fluoroscopic unit (Innova 2100, GE Healthcare, Waukesha, WI, USA). The minimum fluoroscopy dose compatible with adequate imaging will be used.

4.4 Post ablation assessment and follow-up

A 12-lead electrocardiogram (ECG) will be performed for all patients when the patient returned to the ward from the catheter room. Patients will receive a physical examination and ECG prior to discharge. Arrhythmia recurrence will be documented by ECG and patient diary records. In detail, patients will be assessed at 1, 6, 12, 18, 24, and 30 months after RFCA by clinical evaluation with standard ECG and ECG Holter monitoring. An exercise stress test will be performed on consenting patients. Telephone contact will be maintained with patients throughout the entire study to assess the long-term recurrences of symptoms. Recurrence is deﬁned as a relapse of pre-excitation (delta-waves) due to RAPs, ECG-documented tachycardia or return of clinical symptoms that are identical to those before ablation and eventually proved to be AVRT due to RAPs by the following EP study.

4.5 Outcome measures

Outcomes such as the procedure times, fluoroscopy times and procedure success will be used to assess efficiency and efficacy of 3D mapping system on RAPs ablation. Procedural complications will be recorded to assess safety.

**Primary outcomes**

The primary outcome in this trial are the fluoroscopy times and procedural success.

*Fluoroscopy times and procedural success*

The fluoroscopy time is defined as the cumulative duration of fluoroscopy during the entire procedure.

Procedural success is defined as follows: 1) no evidence of RAP conduction, and 2) no related AVRT could be induced for more than 30 minutes after the last radiofrequency energy application, under basal conditions or with intravenous isoprenaline, accompanied by documentation of transient atrioventricular block with adenosine.

**Secondary outcomes**

The secondary outcomes in this study include procedure times, complications, the number of ablation pulses and total ablation time. The preparation time is calculated from the time the patient entered the procedure room until the time of the beginning of needle puncture. The geometry time is measured from the insertion of the first catheter until the beginning of the EP study. The EP study time is defined as the interval from the beginning of initial premature extrastimuli until the definition of the ablation target. The total procedure time is defined as the interval from the placement of the first venous sheath until the removal of the last sheath from the patient.

The number of ablation pulses and total ablation time (i.e., total time that ablation energy was on) will be recorded. These two indicators can reflect the extent of ablation-related damage to the heart.

Complications are deﬁned as permanent second- or third-degree atrioventricular block, vascular or cardiac injury, and pericardial effusion.

4.6 Statistical methods

**Sample size**

Although several studies have investigated the effect of electroanatomical mapping systems on paroxysmal SVT ablation, no RCTs have assessed the effect of 3D mapping systems on RAP ablation. There is no previous study on which to base the sample size calculation. According to non RCTs investigating effects of electroanatomical mapping systems on paroxysmal SVT ablation [6,8-12] and taking into account the minimum number of subjects necessary to evaluate the efficacy of 3D mapping systems on RAP ablation, this pilot study will include about 60 participants with AVRT due to RAP (30 in each group).

**Statistical analysis**

Data will be analyzed by a statistician blinded to the group allocations using the SPSS 10.0 software (SPSS Inc., Chicago, IL, USA). A P-value < 0.05 will be considered statistically significant. Data will be reported as means ± standard deviations (SDs). Student’s t-test, Pearson’s chi-squared test, Fisher’s exact test and correlation analyses, and receiver operator characteristic (ROC) curves will be used where appropriate.

4.7 Patient safety

In this study, any adverse events, include permanent second- or third-degree atrioventricular block, vascular or cardiac injury, pericardial effusion, and other complications related to RFCA treatment will be observed and reported.

1. Quality control

All staff will be required to undergo special training and will be supervised to control bias before participating in the trial. For example, staff will be trained to select participants and to collect data. The monitors will identify problems in the project implementation process in a timely manner and to implement the corresponding countermeasures. Dropouts and withdrawals from the study will be recorded through the intervention and follow-up periods.

1. Protocol amendments and confidentiality

Notification of major modifications to the study (inclusion criteria, outcomes, and analytical method) will be provided to the Ethics Committee, subjects, register, and publisher. The researchers promise to keep the information and technology involved in the files confidential. The personal information on the subjects will be strictly confidential, and not disclosed under any circumstances.

1. Declaration of interest

This study will be supported by the Sun Yat-Sen University Clinical Research 5010 Program (Grant No. 2007011), the National Natural Science Foundation of China (Grant No. 81200173), and the Specialized Research Fund for the Doctoral Program of Higher Education (Grant No. 20120171120078). The funding provided resources to assist with the collection, management, analysis and interpretation of the data, and preparation and review of the manuscript. These academic institutions will provide funding and oversight of funding, but will not be directly involved in collection or cleaning of data, analysis of results, or drafting of the manuscript.

The researchers who take part in the proposed study will not have any conflicts of interests. The researchers will not have any conflicts of interests with the subjects.

1. References

[1] Nishida T, Nakajima T, Kaitani K, Takitsume A, Soeda T, Okayama S, et al. (2012) Non-contact mapping system accurately localizes right-sided accessory pathways in type B Wolff-Parkinson-White syndrome. Europace. 14:752-60.

[2] Lickfett L, Mahesh M, Vasamreddy C, Bradley D, Jayam V, Eldadah Z, et al. (2004) Radiation exposure during catheter ablation of atrial fibrillation. Circulation. 110:3003-10.

[3] Rosenthal LS, Beck TJ, Williams J, Mahesh M, Herman MG, Dinerman JL, et al. (1997) Acute radiation dermatitis following radiofrequency catheter ablation of atrioventricular nodal reentrant tachycardia. Pacing Clin Electrophysiol. 20:1834-9.

[4] Nahass GT. (1995) Fluoroscopy and the skin: implications for radiofrequency catheter ablation. Am J Cardiol. 76:174-6.

[5] Casella M, Pelargonio G, Dello Russo A, Riva S, Bartoletti S, Santangeli P, et al. (2011) "Near-zero" fluoroscopic exposure in supraventricular arrhythmia ablation using the EnSite NavX mapping system: personal experience and review of the literature. J Interv Card Electrophysiol. 31:109-18.

[6] Papagiannis J, Tsoutsinos A, Kirvassilis G, Sofianidou I, Koussi T, Laskari C,  et al. (2006) Nonfluoroscopic catheter navigation for radiofrequency catheter ablation of supraventricular tachycardia in children. Pacing Clin Electrophysiol. 29:971-8.

[7] Schilling RJ, Peters NS, Davies DW. (1998) Simultaneous endocardial mapping in the human left ventricle using a noncontact catheter: comparison of contact and reconstructed electrograms during sinus rhythm. Circulation. 98:887-98.

[8] Smith G, Clark JM. (2007) Elimination of fluoroscopy use in a pediatric electrophysiology laboratory utilizing three-dimensional mapping. Pacing Clin Electrophysiol. 30:510-8.

[9] Volkan Tuzcu A Nonﬂuoroscopic. (2007) Approach for Electrophysiology and Catheter Ablation Procedures Using a Three-Dimensional Navigation System. PACE; 30:519–525

[10] Spar DS, Anderson JB, Lemen L, Czosek RJ, Knilans TK. (2013) Consequence of use of lower dose flat plate fluoroscopy in pediatric patients undergoing ablation for supraventricular tachycardia. Am J Cardio; 112:85-9.

[11] Minglong Chen, Bing Yang, Weizhu Ju, Hongwu Chen, Chun Chen, Xiaofeng Hou, et al. (2010) Right-Sided Free Wall Accessory Pathway Refractory to Conventional Catheter Ablation:Lessons From 3-Dimensional Electroanatomic Mapping. J Cardiovasc Electrophysiol; 21:1317-1324.

[12] Deyong Long, Jianzeng Dong, Caihua Sang, Chenxi Jiang, Ribo Tang, Qian Yan, et al. (2013) Ablation of Left-Sided Accessory Pathways with Atrial Insertion Away from the Mitral Annulus Using an Electroanatomical Mapping System. J Cardiovasc Electrophysiol; 1: 1-5.

Table 4: Time of visits and data collection

|  | **Baseline** | | **RFCA** | **Follow up (months)**  1 6 12 18 24 30 | | | | | | |
| --- | --- | --- | --- | --- | --- | --- | --- | --- | --- | --- |
| **Patients** | | | | | | | | | | |
| Physical examination | × | |  |  | |  |  |  |  |  |
| Medical history | × | |  |  | |  |  |  |  |  |
| Informed consent | × | |  |  | |  |  |  |  |  |
| Randomization | × | |  |  | |  |  |  |  |  |
| **Intervention** |  | |  |  | |  |  |  |  |  |
| 3D group |  | guided by NavX™ system | | |  |  |  |  |  |  |
| Conventional group |  | guided by fluoroscopy system | | |  |  |  |  |  |  |
| **outcomes** |  | |  |  | |  |  |  |  |  |
| Fluoroscopy time (minutes) |  | | × |  | |  |  |  |  |  |
| Acute success rate, (%) |  | | × |  | |  |  |  |  |  |
| procedure time |  | | × |  | |  |  |  |  |  |
| ablation time |  | | × |  | |  |  |  |  |  |
| Complication |  | | × | × | |  |  |  |  |  |
| Recurrence, (%) |  | |  | × | | × | × | × | × | × |
