# Supplementary material for: Catheter Ablation of Right-Sided Accessory Pathways in Adults Using the Three-Dimensional Mapping System: A Randomized Comparison to the Conventional Approach
Source: PLoS One. 2015 Jun 17;10(6):e0128760. doi: 10.1371/journal.pone.0128760 (PMC4471277; doi:10.1371/journal.pone.0128760)
Supplement: S2 Protocol — (DOC) [file pone.0128760.s004.doc]

# EnSite NavX三维标测系统指导下右侧旁路射

# 频消融试验方案

**比较三维标测系统和传统标测系统在右侧旁路射频消融中有效性和安全性**

**的单中心、随机、平行对照临床试验**

| **临床研究申办单位**： | 中山大学附属第一医院 |
| --- | --- |
| **临床研究主要研究者**： | 唐安丽 |
| **临床研究负责单位**： | 中山大学附属第一医院 |
| **数据管理与统计分析**： | 中山大学附属第一医院 |
| **方案版本号**： | 2.0 |
| **方案制订日期**： | 2008年1月1日 |

*本临床试验研究方案属机密资料，为申办者所有。*

*未经申办者许可，不得向第三方（团体或个人）提供部分或全部内容。*

**目 录**

[摘 要 1](#__RefHeading___Toc361076931)

[临床研究流程图 3](#__RefHeading___Toc361076933)

[1． 前言 4](#__RefHeading___Toc361076934)

[2． 研究目的 4](#__RefHeading___Toc361076935)

[3． 研究设计 4](#__RefHeading___Toc361076936)

[3.1总体设计 4](#__RefHeading___Toc361076937)

[3.2 随机化分组 4](#__RefHeading___Toc361076938)

[3.3 样本量与分配 4](#__RefHeading___Toc361076939)

[3.4盲法 5](#__RefHeading___Toc361076942)

[4． 研究人群 5](#__RefHeading___Toc361076946)

[4.1入选标准 5](#__RefHeading___Toc361076947)

[4.2排除标准 6](#__RefHeading___Toc361076948)

[4.3退出（脱落）标准 6](#__RefHeading___Toc361076949)

[4.4剔除标准 7](#__RefHeading___Toc361076950)

[5． 手术干预 7](#__RefHeading___Toc361076953)

[5.1传统方法 7](#__RefHeading___Toc361076954)

[5.2三维方法 8](#__RefHeading___Toc361076955)

[6． 研究过程 9](#__RefHeading___Toc361076960)

[6.1 筛选期（第-3～0天）患者入选 9](#__RefHeading___Toc361076961)

[6.2 访视1（术后1月） 10](#__RefHeading___Toc361076962)

[6.3 访视2（术后6月） 10](#__RefHeading___Toc361076963)

[6.4 访视3（术后12月） 10](#__RefHeading___Toc361076964)

[6.5 访视3（术后18月） 10](#__RefHeading___Toc361076964)

[6.6 访视3（术后24月） 11](#__RefHeading___Toc361076964)

[6.7 访视3（术后30月） 11](#__RefHeading___Toc361076964)

[7． 疗效评价 11](#__RefHeading___Toc361076965)

[7.1主要疗效指标 11](#__RefHeading___Toc361076966)

[7.2 次要疗效指标 11](#__RefHeading___Toc361076967)

[7.3 疗效评定标准 11](#__RefHeading___Toc361076968)

[8. 安全性评价 12](#__RefHeading___Toc361076974)

[8.1不良事件 12](#__RefHeading___Toc361076975)

[8.2实验室检查 12](#__RefHeading___Toc361076976)

[9. 伴随疾病和治疗 12](#__RefHeading___Toc361076977)

[9.1 伴随疾病 12](#__RefHeading___Toc361076978)

[9.2 伴随治疗 12](#__RefHeading___Toc361076979)

[9.3 禁用药物 13](#__RefHeading___Toc361076980)

[10. 不良事件及严重不良事件 13](#__RefHeading___Toc361076981)

[10.1 不良事件的定义 13](#__RefHeading___Toc361076982)

[10.2 严重不良事件的定义 14](#__RefHeading___Toc361076983)

[10.3 缺乏疗效 14](#__RefHeading___Toc361076984)

[10.4 临床检验结果异常和其它异常指标作为不良事件或严重不良事件 15](#__RefHeading___Toc361076985)

[10.5 发现不良事件的方法，频率和时限 15](#__RefHeading___Toc361076986)

[10.6 不良事件和严重不良事件的记录 15](#__RefHeading___Toc361076987)

[10.7 不良事件和严重不良事件的评估 16](#__RefHeading___Toc361076988)

[10.8不良事件和严重不良事件的访视 17](#__RefHeading___Toc361076991)

[10.9 严重不良事件的处理和报告 17](#__RefHeading___Toc361076992)

[10.10 研究结束后的不良事件和严重不良事件 18](#__RefHeading___Toc361076993)

[10.11 与参加试验有关的不良事件和严重不良事件 18](#__RefHeading___Toc361076994)

[10.12 本研究不良反应及处理方法 18](#__RefHeading___Toc361076995)

[10.12.1 本研究常见不良反应及处理方法 18](#__RefHeading___Toc361076996)

[10.12.2不良反应及处理参考方法 18](#__RefHeading___Toc361076997)

[11. 用药依从性 18](#__RefHeading___Toc361076998)

[12. 退出研究 18](#__RefHeading___Toc361076999)

[12.1 受试者完成试验 18](#__RefHeading___Toc361077000)

[12.2 受试者退出试验 19](#__RefHeading___Toc361077001)

[12.3 筛选和基线评估失败 19](#__RefHeading___Toc361077002)

[13. 数据管理 19](#__RefHeading___Toc361077003)

[14. 统计分析 20](#__RefHeading___Toc361077004)

[14.1分析数据集 20](#__RefHeading___Toc361077005)

[14.2 统计方法与内容 20](#__RefHeading___Toc361077006)

[14.3统计软件与一般要求 21](#__RefHeading___Toc361077013)

[14.4期中分析 21](#__RefHeading___Toc361077014)

[15. 研究管理 22](#__RefHeading___Toc361077015)

[15.1 遵从GCP的要求 22](#__RefHeading___Toc361077016)

[15.2 保护受试者的隐私权 22](#__RefHeading___Toc361077017)

[15.3 知情同意书 22](#__RefHeading___Toc361077018)

[15.4 方案的修订程序 22](#__RefHeading___Toc361077019)

[15.5 病例报告表记录及修改程序 22](#__RefHeading___Toc361077020)

[15.6 质量控制和质量保证 23](#__RefHeading___Toc361077021)

[15.7 资料保存 23](#__RefHeading___Toc361077022)

[15.8 项目进度计划 24](#__RefHeading___Toc361077023)

[16. 论文发表 24](#__RefHeading___Toc361077024)

[17. 参考文献 24](#__RefHeading___Toc361077025)

[附件：临床试验方案确认签字页 25](#__RefHeading___Toc361077026)

# 摘 要

| 方案名称 | 比较三维标测系统和传统标测系统在右侧旁路射频消融中有效性和安全性的单中心、随机、平行对照临床试验 |
| --- | --- |
| 适应症 | 右侧旁路介导的阵发性室上速 |
| 试验目的 | 比较三维标测系统和传统标测系统在右侧旁路射频消融中有效性和安全性 |
| 申办单位 | 中山大学附属第一医院 |
| 试验人群 | 符合入选标准且不在排除标准中的患者 |
| 试验样本量 | 计划纳入60例右侧旁路患者，分试验组和对照组，每组约30例， |
| 干预 | （1）所有阵发性室上速患者随机分为三维组和对照组  （2）对照组在常规标测系统指导下行心电生理检查和射频消融术，三维组在电解剖三维标测系统(Ensite NavX™)指导下行心电生理检查和射频消融术。 |
| 入选标准 | （1）>18岁，男女不限；  （2）有阵发性室上速的证据；  （3）所有抗心律失常药至少停用5个半衰期  （4）自愿参加本临床试验，能理解和签署知情同意书。 |
| 排除标准 | 1. 房室结折返性心动过速；左侧旁路介导的阵发性室上速；房   性心动过速；交界性心动过速；窦性心动过速；  （2）结构性心脏病；  （3）合并其它严重疾病；  （4）由药物、电解质紊乱或酸碱平衡失调等因素引起的心律失常  者；  （5）未能控制的高血压，如3级高血压或属高危以上，或严重低  血压者；  （6）未能控制的糖尿病患者；  （7）严重呼吸功能障碍或哮喘者；  （8）原发性造血系统疾病、其他系统疾病（如甲状腺功能亢进等）、  末梢循环灌注不良、严重周围血管疾病及病因不明导致的心律失  常；  （9）过敏体质者、精神病患者、大量饮酒、吸烟者；  （10）妊娠或哺乳期妇女；  （11）近3个月内曾参加其他临床试验者；  （12）研究者认为不适宜入组者。 |
| 随访时间点 | 第1月，第6月，第12月，第18月，第24月，第30月 |
| 疗效指标 | **主要疗效指标**  （1）手术成功率；  （2）透视剂量(透视时间，累积剂量)。  **次要疗效指标**  （1） 手术时间；  （2）消融次数，消融总时间；  （3）并发症； |
| 安全性指标 | 观察并发症发生情况； |
| 统计方法 | 计量资料用均数±标准差（X±SD）表示。t检验用于两组之间定量资料的比较。卡方检验用于定性资料比较。P<0.05为显著性差异具有统计学意义。统计分析采用SPSS软件包10.0处理。 |

#

# 临床研究流程图

|  | 基线 | | 手术干预 | 随访（月）  1 6 12 18 24 30 | | | | | | |
| --- | --- | --- | --- | --- | --- | --- | --- | --- | --- | --- |
| **患者** | | | | | | | | | | |
| 体格检查 | × | |  |  | |  |  |  |  |  |
| 病史 | × | |  |  | |  |  |  |  |  |
| 知情同意 | × | |  |  | |  |  |  |  |  |
| 随机化 | × | |  |  | |  |  |  |  |  |
| **干预** |  | |  |  | |  |  |  |  |  |
| 3D 组 |  | NavX™ 标测 | | |  |  |  |  |  |  |
| 传统组 |  | 传统标测 | | |  |  |  |  |  |  |
| **观察指标** |  | |  |  | |  |  |  |  |  |
| 透视时间 (分钟) |  | | × |  | |  |  |  |  |  |
| 手术成功率, (%) |  | | × |  | |  |  |  |  |  |
| 手术时间 |  | | × |  | |  |  |  |  |  |
| 消融时间 |  | | × |  | |  |  |  |  |  |
| 并发症 |  | | × | × | |  |  |  |  |  |
| 复发, (%) |  | |  | × | | × | × | × | × | × |

前言

**1.1背景介绍**

右室旁路的射频消融治疗由于缺乏标测导管指引三尖瓣环位置、消融电极贴靠稳定性差、前间隔旁道过于靠近希氏束及心外膜旁道发生率高等原因，传统方法下进行射频消融治疗难度相对较大，成功率较低。NavX系统是最常用的接触式标测系统之一，它能通过使用普通电生理导管及体表贴片重构目标心腔的解剖学模型并在模型上反映心腔内每个稳定心动周期中电激动的扩布，从而指导多种心律失常的射频消融治疗。NavX三维标测系统已经广泛用于房颤和室速的射频消融，但在右室旁路介导的阵发性室上速中的应用情况研究很少。本研究旨在比较NavX三维标测系统指导下与传统方法（单纯X线透视）指导下的右室旁路射频消融治疗，旨在探讨三维标测系统在右室旁路射频消融中的临床应用价值。

**1.2 临床研究**

本试验为单中心，随机，平行对照研究。本实验拟在中山大学附属第一医院（三级甲等医院）心血管内科完成，研究期限定为2011年6月到2013年11月。所有入选患者在入组前需签署知情同意书。入组患者将会随机分到3D 组和传统手术组(图1).. 病人将在手术当天和术后1月，6月，12月，18月，24月和30月接受随访，随访方法为普通心电图或动态心电图检查（表1）。分析结果的研究人员不知道两组患者的处理情况。本研究已在中国临床试验注册中心进行注册，该中心也是世界卫生组织国际临床试验注册平台一级注册机构。

**2. 研究目的**

比较三维标测系统和传统标测系统在右侧旁路射频消融中有效性和安全性。

3. **研究设计**

## 3.1总体设计

本研究遵循GCP原则，开展单中心、随机、统计盲法、平行对照的设计方法。

## 3.2 随机化分组：

随机化的实施应用SAS软件由计算机产生随机排列表的方法进行随机区组设计，区组大小为4。

## 3.3 样本量与分配

### 3.3.1样本量

尽管已研究探讨过电解剖标测系统在阵发性室上速中的作用，但据我们所知尚无随机临床试验研究过三维标测系统在右室旁路中的作用，因此无法根据既往试验推测和计算样本量。我们根据临床试验所需的最小样本量以及既往非随机临床试验的文献拟在本研究中纳入60例右室旁路介导的阵发性室上速患者，试验组和对照组各约30例。

### 3.3.2 病例分配

将符合入选标准，且不在排除标准中的病例作为受试者。进入试验的病例，按先后次序发给标有相应编号的信封，不得任意选择试验编号。

## 3.4盲法

### 3.4.1 盲法设计

该试验无法采用双盲设计，受试者和手术者都知道干预方法是试验组还是对照组，但数据收集人员和统计分析人员采用盲法，不知道患者干预方法是试验组还是对照组。

### 3.4.2编盲和盲底保存

本研究开始前，由申办单位（或合同研究组织）会同统计人员进行统一编盲。由本次研究中的统计人员，在计算机上利用统计软件，按分层区组随机的方法产生随机编码表。由与本临床研究的临床观察、监查无关的人员，根据已形成的随机处理编码将相应的编号放在不透明的信封中。

产生的全部处理编码，包括编码所对应的组别、产生随机编码的种子数等参数所形成的盲底密封起来，交由申办单位和临床研究负责单位保存，该盲底应妥善保存至临床研究结束后。

全部编盲过程将记录并书写成文件形式，即编盲记录，作为本次临床研究的重要文件之一保存。其内容包括：随机处理编码的产生，每个受试者编号，干预方式，盲底的保存，揭盲的规定等。

### 3.4.3揭盲规定

本研究采用二次揭盲法，当编号信封打开后确定患者为三维手术组或传统手术组时，为第一次揭盲当。当数据全部录入数据库，并经答疑、核查、盲态审核（Blind Review）并确认最终的统计计划书后，数据库将被锁定（Locked），随后对全部数据进行统计分析，统计分析完成后对数据收集和统计计算人员进行第二次揭盲。所有揭盲过程均应有记录。

# 4. 研究人群

## 4.1入选标准

符合下列所有条件的受试者进入本试验：

（1）>18岁，男女不限；

（2）有阵发性室上速的证据；

（3）所有抗心律失常药至少停用5个半衰期；

（4）自愿参加本临床试验，能理解和签署知情同意书。

## 4.2排除标准

**有下列情况之一者不能入选本试验：**

（1）房室结折返性心动过速；左侧旁路介导的阵发性室上速；房

性心动过速；交界性心动过速；窦性心动过速；

（2）结构性心脏病；

（3）合并其它严重疾病；

（4）由药物、电解质紊乱或酸碱平衡失调等因素引起的心律失常

者；

（5）未能控制的高血压，如3级高血压或属高危以上，或严重低

血压者；

（6）未能控制的糖尿病患者；

（7）严重呼吸功能障碍或哮喘者；

（8）原发性造血系统疾病、其他系统疾病（如甲状腺功能亢进等）、

末梢循环灌注不良、严重周围血管疾病及病因不明导致的心律失

常；

（9）过敏体质者、精神病患者、大量饮酒、吸烟者；

（10）妊娠或哺乳期妇女；

（11）近3个月内曾参加其他临床试验者；

（12）研究者认为不适宜入组者。

**4.3退出（脱落）标准**

因以下原因未完成临床方案的入组病例应视为脱落：

（1）临床试验中受试者发生了某些合并症、并发症或特殊生理变化，不适宜继续进行研究者；

（2）受试者依从性差，影响安全性和疗效评价者；

（3）发生不良事件或严重不良事件，不适宜继续接受试验的受试者；

（4）受试者不愿意继续进行临床试验，主动提出退出者；

（5）失访；

（6）研究者判断需要退出试验的其他情况。

处理原则：

对于中途退出试验病例或失访脱落病例，研究者应积极采取措施，尽可能完成最后一次检测，以备对其疗效和安全性进行分析。

所有脱落病例，均应在病例报告表中，填写试验结论表及病例脱落的原因。一般情况下有几种，即发生不良事件、违背试验方案（包括依从性差）、失访（包括受试者自行退出试验）、被申办者中止和其他。

## 4.4剔除标准

（1）病例入选后，发现不符合纳入标准或符合排除病例标准者；

（2）病例入选后未行射频消融手术者；

（3）病例入选后即自动脱落失访，无疗后访视记录者。

剔除的病例应说明原因，病例资料应保存完整备查。不作疗效统计分析，但至少接受一次

治疗，且有安全性记录者，视情况可参加安全性分析。

### 4.5中止标准

### 指临床试验尚未按方案结束，中途停止全部试验。试验中止的目的主要是为了保护受试者权益，保障试验质量，避免不必要的经济损失。

（1）试验过程中发现所定试验方案有重大失误或操作过程中发现重大偏差，难以对药物进行评价；

（2）申办者因经费或管理等原因要求中止试验；

（3）国家管理部门因某种原因勒令中止试验。

# 5. 手术干预

**5.1传统方法下手术过程**

5.1.1电极放置 选择右股静脉及右颈静脉（或锁骨下静脉）作为穿刺入路，Seldinger法穿刺血管后，在X线透视辅助下分别置入四极电极至右心室，十极电极至冠状静脉窦内。然后，再次穿刺右股静脉，置入大头电极或另一个标测电极。

5.1.2电生理检查 分别在心房、心室至少在两个不同部位进行程序刺激。心室程序刺激分别在右室基底部及右室流出道进行S1S1间期递减刺激，S1S2早搏刺激，必要时早搏刺激可增加至S5。心房程序刺激分别在高位右心房及冠状静脉窦（窦内A波最大的导联）进行S1S1间期递减刺激，S1S2早搏刺激，及burst快速起搏刺激。必要时静滴异丙肾上腺素在心率增加25%-40%后重复上述检查。电生理检查移动电极至不同位置刺激均在X线透视指导下完成。

1.3.3靶点标测及射频消融 隐匿性旁道在心室起搏或OAVRT发作时沿三尖瓣环进行标测，寻找三尖瓣环处心房最早激动点，通过多导电生理记录仪观察腔内电图VA融合最紧密处定为靶点，并利用X线透视确认大头电极位置及观察大头贴靠的稳定性；若为无逆传功能的显性旁道则在窦性心律下标测三尖瓣环处心室激动最早点作为消融靶点。

**5.2 NavX系统导航下手术过程**

5.2.1 电极放置 放置的电极类型及选择的静脉入路与传统方法相同。放置冠状窦电极利用NavX系统的点云功能，记录冠状窦电极的轨迹，当冠状窦电极首先出现心房电位处，为上腔静脉与右房交界，电极向下腔静脉方向移动，至心房电位消失处为下腔静脉与右房交界处，回撤电极，将电极送至右室，记录到心室电位而无心房电位时顺时针旋转电极同时缓缓回撤到右房室环处（A、V振幅大致相等处）并递送至冠状窦内（如图1）。放置右心房/右心室电极及大头电极同样利用点云功能指引导管到位。如导管递送时有困难或单个导管10分钟内不能到位则在X线透视下进行。

5.2.2电生理检查 电生理检查方案与传统方法相同，但均在NavX系统指导下根据电生理检查需要调整电极位置，除非电极到位困难，否则不使用X线透视。

5.2.3 靶点定位及消融 利用NavX系统的OneMap功能同时进行建模和激动标测，右心腔建模包括上腔静脉、下腔静脉、右房心腔、三尖瓣环及标注希氏束和冠状窦口位置。在建立三尖瓣环模型时，我们将其构建成类似12点时钟钟面结构，先标记出His束位置，定为2点，冠状窦口定为5点，上腔静脉、下腔静脉分别是12点和6点，最右侧游离壁位置为9点，如图 2。在心室起搏时或顺向型心动过速发作时进行激动标测，寻找心房逆传最早激动点进行消融，如图3；若旁道只有前传功能，则在窦律下标测心房侧V波最早出现的位置作为靶点。如消融过程出现贴靠不稳定则利用SR0鞘辅助，在X线透视下递送SR0鞘管，SR0鞘进入右心房后置入大头电极，大头电极送出鞘管以后通过NavX系统观察大头走形从而调整SR0鞘管。消融终点为旁道前传及逆传功能均被阻断，重复完整电生理程序刺激不能诱发室上性心动过速发作。

# 6. 研究过程

## 6.1 筛选期：（第-3～0天）患者入选

（1）签署知情同意书；

（2）核查入选、排除标准；

（3）取得病史和人口学资料；

（4）生命体征检测和体格检查；

（5）疗效观察和记录；

（6）妊娠试验；

（7）实验室检查：

血常规（红细胞计数、血红蛋白、白细胞计数、血小板计数、中性粒细胞百分比、淋巴细胞百分比）

尿常规（尿pH、尿蛋白、尿红细胞、尿白细胞）

肝功能（ALT、AST、TBIL）

肾功能（Cr、BUN）

血脂（TC、TG、HDL-C、LDL-C）

血糖

凝血四项（凝血酶原时间（PT）、活化部分凝血活酶时间（APTT）、凝血酶时间（TT）、纤维蛋白原（FIB））

电解质（钾、钠、氯离子）

血尿酸

hs-CRP

术前筛查组合（HIV, 梅毒，肝炎系列）

（8）12导联心电图；

（9）心脏彩超（左室射血分数（LVEF）、E/A比值、心脏指数（CI）、心排血量（CO）、每搏量（SV）、左心室舒张末内径（LVEDD）、心肌作功指数（Tei 指数））；

（11）随机入组；

（12）接受手术；

（13）术后评估:体格检查，12导心电图，心脏彩超。

（14）预定下次访视时间；

## 6.2 访视1（术后1月）

（1）生命体征和体格检查；

（2）疗效观察和记录；

（3）12导心电图或动态心电图；

（4）记录不良事件；

（5）预约下次访视时间；

## 6.3 访视2（术后6月）

（1）生命体征和体格检查；

（2）疗效观察和记录；

（3）12导心电图或动态心电图；

（4）记录不良事件；

（5）预约下次访视时间；

## 6.4 访视3（术后12月）

（1）生命体征和体格检查；

（2）疗效观察和记录；

（3）12导心电图或动态心电图；

（4）预约下次访视时间；

## 6.5 访视4（术后18月）

（1）生命体征和体格检查；

（2）疗效观察和记录；

（3）12导心电图或动态心电图；

（4）预约下次访视时间；

## 6.6 访视5（术后24月）

（1）生命体征和体格检查；

（2）疗效观察和记录；

（3）12导心电图或动态心电图；

（4）预约下次访视时间；

## 6.7 访视6（术后30月）

（1）生命体征和体格检查；

（2）疗效观察和记录；

（3）12导心电图或动态心电图；

（4）预约下次访视时间；

# 7.疗效评价

# 7.1主要疗效指标

（1）手术成功率；

（2）透视剂量(透视时间，累积剂量)。

7**.2 次要疗效指标**

（1） 手术时间；

（2）消融次数，消融总时间；

（3）并发症；

**7.3 疗效指标测定方法**

**手术时间和透视时间**

准备时间指患者从进入导管室开始算起到穿刺开始的时间。建模时间指从插入第一根导管开始到开始心内电生理检查的时间。电生理检查时间指从第一个期前刺激开始算起到消融靶点确定为止的时间。总的手术时间定义为从置入第一根静脉导管开始到拔出最后一根静脉导管的时间。消融总次数和消融总时间可直接从射频仪上读出和记录。透视时间指在整个手术过程中接受到放射线照射的时间。放射剂量指整个手术过程中患者接受的累及辐射剂量。

**手术成功与并发症**

手术成功定义如下: 1) 无右侧旁路存在的证据 2)无论在基础条件还是在异丙肾上腺素刺激情况下无法诱发心动过速，同时使用腺苷后出现一过性房室传导阻滞。

手术并发症主要包括：血管损伤，房室传导阻滞，心脏损伤，心包填塞等。

# 8. 安全性评价

## 8.1不良事件

记录整个试验过程中受试者发生的任何未预期或不适的症状、体征、疾病或可能导致身体伤害，暂时与手术干预有关联，但不一定与手术干预有因果关系的事件。

## 8.2实验室检查

于手术前进行实验室检查，内容如下：

血常规（红细胞计数、血红蛋白、白细胞计数、血小板计数、中性粒细胞百分比、淋巴细胞百分比）

尿常规（尿pH、尿蛋白、尿红细胞、尿白细胞）

肝功能（ALT、AST、TBIL）

肾功能（Cr、BUN）

血脂（TC、TG、HDL-C、LDL-C）

血糖

凝血四项（凝血酶原时间（PT）、活化部分凝血活酶时间（APTT）、凝血酶时间（TT）、 纤维蛋白原（FIB））

电解质（钾、钠、氯离子）

血尿酸

hs-CRP

术前筛查组合（HIV, 梅毒，肝炎系列）

研究开始前需获得相关实验室检查项目的正常值范围。随防过程中如研究者认为有必要也可行相关检查。

# 9. 伴随疾病和治疗

## 9.1 伴随疾病

在知情同意书给予时出现的疾病将被认为是伴随疾病，将被记录在病例报告表上。

## 9.2 伴随治疗

所有在受试者进入研究时或研究期间任何时间进行的其它治疗被认为是伴随治疗，所用药品以通用名的形式记录在病例报告表中。试验期间的伴随用药必须是受试者所必需使用的，研究者若认为对试验药物无干扰作用方可酌情决定是否给予，剂量应保持在最低水平。

因某些疾病不得不在试验期间使用时，除符合以上原则，应注意对给药剂量的控制。

## 9.3 禁用药物

随访期间禁止使用以下药物：

抗心律失常药物的西药和中药，具体抗心律失常药包括：

1. 钠通道阻滞药，如奎尼丁、普鲁卡因胺、普罗帕酮、氟卡尼等；
2. β肾上腺素受体拮抗药，如普萘洛尔、比索洛尔、阿替洛尔等；
3. 延长动作电位时程药，如胺碘酮等；
4. 钙通道阻滞药，如维拉帕米和地尔硫等；
5. 中药，如稳心颗粒、参松养心颗粒等。

# 10. 不良事件及严重不良事件

研究者有责任发现并记录符合本研究方案规定的不良事件和严重不良事件定义的事件。

## 10.1 不良事件的定义

不良事件是指发生在使用某种药物或接受某种治疗的患者或临床研究对象身上的任何一件不利的医疗事件，它与此种治疗不一定有因果关系。

因此不良事件可以是任何不利的和未预料到的体征（包括有临床意义的实验室检查结果异常）、症状或与应用药物暂时相伴的疾病（新出现的或原有疾病的恶化）；如果是市售药品，还应包括使用后未产生预期疗效（即缺乏疗效）、滥用和误用。

不良反应包括：

（1）原有疾病出现明显的或未预见的加重或恶化；

（2）原有的慢性疾病恶化或间断发作性疾病加重，表现为频率和/或强度增加；

（3）在使用研究用药后发现或诊断的病情，即使它在研究开始前可能已经存在；

（4）怀疑相互作用而出现的体征、症状或临床后遗症；

（5）与治疗相关的体征、症状或后遗症。

不良事件不包括：

（1）内科和外科检查治疗过程（如内镜检查、阑尾切除术），但导致需作这些检查的疾病是不良事件；

（2）有害的医疗事件并未发生的情况（如因社会收容或方便病人而收入院）；

（3）在研究开始时存在或发现的原有疾病或病情出现预期的周期性波动、但并未恶化；

（4）正进行研究的疾病或紊乱，或者与疾病或紊乱相伴的预期进展、症状或体征，除非它们比受试者所患疾病的预期更为严重。

## 10.2 严重不良事件的定义

严重不良事件是指发生于任一组别的符合以下任何条件的不良事件：

（1）导致死亡

（2）危及生命

注：危及生命是指此事件出现时受试者有死亡的危险。这个定义不包括如果病情进一步加重后可能导致死亡的事件。

（3）入院治疗或住院时间延长

注：一般而言，“住院”是指受试者不适合在门诊或急诊观察或处理，而需正式入院或急诊留观（通常至少要过夜）。住院期间出现的合并症为不良事件。如果因合并症导致住院时间延长或达到其它任何严重不良事件的标准，也属于严重不良事件。如果因为既往疾病而进行的择期手术且此疾患较基线时未加重，则不属于严重不良事件。

（4）残疾

注：残疾是指某人正常生活能力的实质性丧失，不包括有关的细小的病症：如单纯性头痛、恶心、呕吐、腹泻、流行性感冒、意外损伤（如踝关节扭伤）等，尽管它们可能对日常生活能力有一定影响，但并不是实质的（长期）丧失。

（6）某些需要医学或科学的判断决定在此种情形下是否紧急报告的情况：虽不会导致死亡、

立刻危及生命或住院治疗但对受试者可能造成危害、或可能需要内科或外科治疗以阻止上述定义所列后果之一发生的重要的医疗事件，则可认为是一种严重不良事件。此种医疗事件包括浸润性或恶性癌肿、需在急诊室或医院监护病房给予重症监护的心脏填塞，心肌梗塞，血管破裂，心脏穿孔等。

## 10.3 缺乏疗效

缺乏疗效本身不能作为不良事件报告，任何缺乏疗效导致的症状/体征或后遗症只有符合不良事件/严重不良事件标准，才需报告。

## 10.4 临床检验结果异常和其它异常指标作为不良事件或严重不良事件

某些异常的实验室检查结果（如临床生化、血液学、尿液分析）或其它异常指标（如心电图、生命体征等）经研究者判定具有临床意义，如果它们符合10.1部分不良事件的定义（“不良事件的定义”），或10.2部分的严重不良事件定义（“严重不良事件的定义”），则必须将其记录为不良事件或严重不良事件予以记录。在治疗后发现的，或者在基线评估时存在并在研究开始后加重的有临床意义的实验室检查结果异常及其它异常所见，则应作为不良事件或严重不良事件。但是，与所研究疾病有关的有临床意义的异常实验室检查结果或其它异常所见，除非研究者判定较所预料的受试者的病情更为严重，否则不包括在不良事件或严重不良事件中。在研究开始时存在或被发现但未加重的异常实验室检查结果或其它异常所见，也不包括在不良事件和严重不良事件中。

由研究者来决定一项异常实验室检查结果或其它异常所见是否具有临床意义。

## 10.5 发现不良事件的方法，频率和时限

不良事件将通过口头提问的方式获悉并记录于CRF不良事件页。研究者或指定人员每次询问不良事件时应使用相同的问题，以免造成受试者间的差异。研究者可以问：

“上次访视以来你是否感觉有什么不同？”

针对受试者对上述提问的回答，研究者可根据患者特殊主诉再提些相关问题，如：

不适症状有多严重？

多长时间出现一次？

一般症状会持续多久？

研究者还需询问受试者关于以往未解决的不良事件的情况。研究者要评估不良事件的强度、严重程度、与治疗的相关性以及处理措施。

从试验开始前至试验结束或相应的退出访视，研究者均要询问受试者上述问题。

## 10.6 不良事件和严重不良事件的记录

发生不良事件/严重不良事件时，研究者有责任回顾所有相关记录（如病程记录、实验室检查和诊断报告），并将与事件有关的资料记录于受试者的病例报告表中。

研究者应尽量依据症状、体征和/或其它临床资料就事件作出判断。在此情况下，诊断应作为不良事件和/或严重不良事件进行记录，而不是患者的体征/症状。

## 10.7 不良事件和严重不良事件的评估

### 10.7.1 严重程度的判定

在研究期间，研究者应依据自己的临床判断，对报告的每起不良事件和严重不良事件的严重程度作出评估。记录在病例报告表中不良事件和严重不良事件应该按下列标准归类：

轻度：只引起受试者轻微不适，不影响日常活动，受试者比较容易耐受的事件；

中度：引起受试者明显不适并妨碍了正常日常活动的事件；

重度：导致无法进行正常日常活动的事件。

注意不要混淆重度的不良事件与严重不良事件：重度是用来衡量事件严重程度的一个类别，不良事件和严重不良事件都可以评为重度，凡符合10.2中“严重不良事件”定义的事件，都应列入严重不良事件。

### 10.7.2 因果关系的判断

研究者必须依据临床经验判断每起不良事件/严重不良事件与所接受治疗之间的关系。按与肯定有关、很可能有关、可能有关、可能无关、无关五级来评定不良事件与治疗之间的关系。

（1）肯定有关：治疗及反应发生时间顺序合理；同时有文献资料佐证；并已排除原患疾病等其他混杂因素的影响；

（2）很可能有关：同“肯定”或虽然有合并其它治疗，但基本可排除合并治疗导致反应发生的可能；

（3）可能有关：治疗与反应发生时间关系密切，同时有文献资料佐证；但引发不良反应的治疗不止一种，或原患疾病病情进展因素不能除外；

（4）可能无关：不良反应与治疗时间关系不密切，反应表现与已知该治疗的不良反应不相吻合；

（5）无关：不良反应与治疗之间没有关系。

不良反应是指1、2和3之和。

其它原因，如潜在疾病的自然病史、同时进行的其它治疗、危险因素以及暂时与所研究治疗有关的事件均需考虑和检查。研究者还应查阅临床研究者手册和/或治疗方案（的相关资料用以作出评估。

研究者在最初报告出现某种严重不良事件时，可能只有很少的信息，然而，在向上呈报严重不良事件的CRF之前，研究者对每一事件进行因果关系评估十分重要。研究者可以根据访视资料更改因果关系的判断并对相应的CRF页进行相应修改。因果关系的判断是必须的指标之一。

研究者应依照CRF中SAE表的格式提供因果关系评估。

## 10.8不良事件和严重不良事件的访视

一旦报告发生不良事件/严重不良事件，研究者应对每一个受试者进行访视，并向申办者提供该事件的访视信息。对所有在前次访视/复诊中已经记录的并持续存在的不良事件/严重不良事件，应在此次的访视/复诊中进行复查。

所有不良事件/严重不良事件都必须访视至其缓解、病情稳定、事件原因另有其它解释或受试者失访。问题一旦得到解决，CRF中不良事件/严重不良事件部分应得到及时更新。研究者可以在访视中增加另外的一些检查，这可能有助于阐明不良事件或严重不良事件的本质和/或原因。这可能包括另外的实验室检查或研究、病理学检查或其它专业人士的会诊。

申办者可要求研究者完成或安排另外增加的可能有助于阐明不良事件或严重不良事件的本质和/或原因的检查和/或评估，研究者有义务予以协助。如果受试者在参加研究期间或在被认可的访视期间发生死亡，应向申办者提供包括组织病理学检查在内的尸检结果。

新的或更新后的资料应被记录于初始完成的CRF严重不良事件页上，研究者应对新的或更新后的资料签名并注明日期。更新的CRF严重不良事件页应按照10.9中规定的时间呈报申办者。

## 10.9 严重不良事件的处理和报告

研究者一旦意识到受试者发生了严重不良事件，必须立即采取治疗措施并在24小时内报告申办者。严重不良事件报告表应尽可能完整、详细地记录所有能得到的与事件有关的资料，由研究者（或指定人）签名，并在规定的时间内呈送申办者。即使研究者未能得到有关严重不良事件的全部资料，也不必等到其它资料完备后才完成CRF相关页，才报告申办者。严重不良事件的CRF页可在获得其它资料后再更新。

在初始报告中，研究者就应依据10.7.2（因果关系的判断）进行因果关系的评估。以传真发送严重不良事件报告表是将此资料呈送至研究项目负责人的最佳方法。在极少数情况下和没有传真设备的条件时，可采取电话通知，第二天寄送严重不良事件报告表复印件。但电话通知不能取代研究者完成并签署严重不良事件报告表。

## 10.10 研究结束后的不良事件和严重不良事件

研究结束后的不良事件/严重不良事件是指任何发生在10.5中（发现不良事件和严重不良事件的方法、频率和时限）规定的不良事件/严重不良事件访视期限以外的任何事件。研究者不必主动探求发生在已结束临床研究和访视的受试者身上的不良事件和严重不良事件。但是，如果研究者在一名受试者已脱离研究后的任何时候知悉了发生了任何严重不良事件（包括死亡），而且此事件有可能与研究治疗有关，研究者应迅速通知申办者。

## 10.11 与参加试验有关的不良事件和严重不良事件

被认为与参加研究有关的严重不良事件（如操作、创伤性检查，现有治疗方案的改变），无论它发生在治疗前还是治疗后，都应迅速报告给申办者（见10.9部分“严重不良事件迅速报呈申办者”）。

## 10.12 本研究不良反应及处理方法

## 10.12.1 本研究常见不良反应及处理方法

本研究中射频消融手术不良反应较少，偶见动静脉瘘、心包填塞等

## 10.12.2不良反应及处理参考方法

**（1）心包填塞**

一旦发生心包填塞立即行心包穿刺抽液，必要时可置管引流或外科开胸手术。本研究术者是有着二十余年电生理手术经验的高年资主任医师，发生不良反应或并发症的可能性小，一旦发生可熟练进行心包穿刺、引流等临床处理，且和我院心脏外科已形成良好的合作机制有完备的流程，一旦需要心脏外科医生10分钟内可至手术室进行急诊手术。

**（2）穿刺部位血肿或动静脉瘘**

通过按压和加压包扎一般可治疗，必要时血管外科手术治疗。

# 11. 随访依从性

在试验结束后，对患者的随访依从性进行评价，记录。

# 12. 退出研究

## 12.1 受试者完成试验

受试者按照试验要求完成计划安排的每次访视规定的所有内容，则视为完成试验。

## 12.2 受试者退出试验

任何进入试验的受试者（已签署知情同意书）由于任何原因未能按照上述定义的要求完成试验, 将被视为退出试验。

每个受试者均可在研究的任何阶段、任何理由（特殊或非特殊）退出该项研究而不受到歧视。患者的治疗将不会受到限制，在适当的情况下，研究者将使用常规的治疗方法为患者治疗。

应尽可能的访视退出试验的患者。随机后退出的患者在计划访视时退出应完成当次访视。所有随机后退出试验的受试者，均应尽量完成访视3要求的所有评估。

参加随机的所有受试者的病例报告表均要填写完整，并且在病例报告表的“试验总结”上记录所有被随机但未完成试验的受试者退出原因。此外，在“试验完成情况总结”上必须记录提前退出试验的受试者末次随访日期。试验结束后有关受试者的所有数据都应当提交给申办者。

## 12.3 筛选和基线评估失败

患者在随机之前退出试验被视为筛选失败。退出试验的原因需记录在患者的筛选/入选表中。

# 13. 数据管理

（1）研究者根据受试者的原始观察记录，将数据及时、完整、正确、清晰地载入病例报告表。

（2）监查员监查试验的进行是否遵循试验方案。确认所有病例报告表填写正确完整，并与原始资料一致。如有错误和遗漏，及时要求研究者改正。修改时需保持原有记录清晰可见，改正处需经研究者签名并注明日期。

（3）经过监查员检查后的病例报告表，由监查员核查签字后，及时送交临床试验数据管理员。对于完成的病例报告表在研究者、监查员、数据管理员之间的传送应有专门的记录，收到时应有相应的签名，记录需妥善保存。

（4）数据管理员在数据录入前再次核查，发现问题及时通知监查员，要求研究者作出回答。他们之间的各种疑问及解答的交换应当采用疑问表形式，疑问表应保存备查。

（5）数据管理员在进行数据录入前，要了解观察表格各项目的内容及编码情况，将编码工作过程记录于编码本保存。数据库命名应规范、易读、易查找。并保证其正确、安全和保密。

（6）数据录入员录入数据采用二次录入。录入过程发现问题或意外情况，应做好登记并及时报告，以便迅速处理问题，数据录入结束后应抽查部分观察表格，了解录入质量，分析并处理存在的问题。

（7）数据管理员应与主要研究者一起，按病例报告表中各指标数值的范围和相互关系拟定数据范围检查和逻辑检查内容。并编写相应的计算机程序，在输入前控制错误数据输入，找出错误原因加以改正，所有错误内容及修改结果应有记录并妥善保存。

（8）原始病例报告表在按要求完成数据录入和核查后，按编号的顺序归档保存，并填有检索目录等，以备查考。电子数据文件包括数据库、检查程序、分析程序、分析结果、编码本和说明文件等，应分类保存，并有多个备份保存于不同磁盘或记录介质上，妥善保存，防止损坏。所有原始档案应按我国《临床试验质量管理规范》的规定期限保存。

# 14. 统计分析

统计分析详见“统计分析计划书”，在数据库锁定前，与统计人员将和主要研究者、申办者一同讨论，根据数据特征定稿，本方案仅提供统计学常规要求。

## 14.1分析数据集

（1）全分析集（FAS）：所有经随机化分组，接受治疗且至少一次具有治疗后评价数据的病例集合。主要疗效指标缺失时，根据意向性分析（intention to treat，ITT分析），用前一次结果结转。FAS为主要分析集。

（2）符合方案集（PPS）：指符合纳入标准、不符合排除标准、完成治疗方案的病例集合，即对符合试验方案、依从性好、完成CRF规定填写内容的病例进行分析（PP分析）。PP分析主要用于主要疗效指标。

（3）安全数据集（SS）：接受治疗，且有安全性指标记录的实际数据。安全性缺失值不得结转；纳入可作评价的部分剔除病例，如年龄超过纳入标准的病例，但不包括使用禁用药物导致无法作安全性判断的病例。不良反应的发生率以安全集的病例数作为分母。

## 14.2 统计方法与内容

### 14.2.1病例入组分析

- 列出中心入选及完成病例数，确定三个分析数据集（FAS，PPS，SS）。
- 列出脱落与剔除病例及其原因。

### 14.2.2人口学资料及基线分析

描述性统计人口学资料及其他基线特征值：

- 连续变量计算其例数、均值、标准差、中位数、最小值和最大值。
- 计数和等级资料计算频数及构成比。
- 推断性统计结果（P值）作为描述性结果列出。

### 14.2.3疗效分析

### 主要疗效指标

（1）手术成功率，采用Pearson卡方或Fisher精确检验比较组间差异。

（2）透视剂量(透视时间，累积剂量)，采用 Student’s T检验。

### 次要疗效指标

（1）手术时间，采用Pearson卡方比较组间差异；

（2）消融次数，消融总时间，采用Pearson卡方比较组间差异；

### 14.2.4安全性分析

- 计算不良事件和不良反应发生率；
- 分系统列出不良事件和不良反应发生的频率和频数，计算百分比；
- 各种不良事件病例的详细列表；
- 各种不良反应病例的详细列表；
- 实验室检查、心电图、体检在试验后“正常转异常”或“异常加剧”的例数和转异率；
- 列出实验室检查、心电图、体检异常病例和临床解释。

## 14.3统计软件与一般要求

- 采用SAS 9.2软件分析；
- 所有的统计检验均采用双侧检验，P值小于或等于0.05将被认为所检验的差别有统计学意义；
- 详细的统计方法将在统计分析计划中提供。

## 14.4期中分析

本研究不进行期中分析。如试验中发生特殊情况，如疗效不确切或安全性问题，将由申办者、研究者和统计专家一同讨论决定。

# 15. 研究管理

## 15.1 遵从GCP的要求

应按照中国药品临床试验管理规范（GCP）的要求来进行本试验。

## 15.2 保护受试者的隐私权

在填写和管理病例报告表时，应考虑到保护患者的隐私，如以患者的随机号来代表患者等，同时可将患者名字的缩写当作其名字的代称。

## 15.3 知情同意书

在开始本试验前，试验人员必须将受试者知情同意书以患者能理解的方式向每一位参加本试验的患者予以解释说明，并获得患者自愿参加本项试验的书面形式的知情同意书。在CRF

中填写获得书面知情同意书的日期。

（1）研究的性质和目的；

（2）研究过程；

（3）受益和风险；

（4）其他的替代治疗；

（5）受试者权利：患者拒绝参加本项试验或在试验进展的任何时候都可以退出本试验，患者的权益不会受到任何影响；

（6）保密协定；

（7）患者必须遵守的事项；

（8）以及认为可以保护患者权益的任何事项。

## 15.4 方案的修订程序

当必须修改方案或暂停本项试验时，主要研究者应同申办者进行联系和磋商，如果决定修改方案而暂停试验时，参加试验的主要研究者应立即将详细情况及原因通知所有参加试验的人员。如果是重大修改，应取得伦理委员会批准后实施。

## 15.5 病例报告表记录及修改程序

真实、准确的记录试验数据。

如需做任何修改，请保持修改前的记录清晰可辨，并署明修改日期。如修改范围较大或改动较大，则修改者应注明修改日期及修改的理由，同时签名。

## 15.6 质量控制和质量保证

（1）采用标准操作规程，以保证临床试验的质量控制和质量保证系统的实施。

（2）为保证试验的质量，在正式试验开始前，由参加试验的核心成员，共同讨论、制定临床研究方案。对参加试验的有关医务人员进行同期培训。

（3）按统一的临床研究方案实施试验。

（4）临床试验中所有观察到的结果和异常发现，均应及时加以认真核实、记录，保证数据的可靠性。临床试验中各种检查项目所使用的各种仪器、设备、试剂、标准品等，均应有严格的质量标准，并确保是在正常状态下工作。临床数据的记录和转移，必须由有经验的医师负责，并有专人监督或核对，以保证数据的科学性和准确性。临床试验的各种结论，必须来源于原始数据。

（5）负责试验的医师，应完整、详细、准确、及时地填写病例报告表（CRF）。交上级医师签名确认后按规定程序报送或保存。所有与试验有关的数据资料应集中管理与分析。

（6）建立数据保管、数据传递、数据查询的程序。保管的资料包括：受试者的原始病历、影像学资料、CRF、受试者筛选表、受试者鉴认代码表、严重不良事件报告表、各医院需填报的GCP表格、访视报告表及有关的各种原始医疗文件等。传递的数据包括：受试者总随机表、CRF、严重不良事件报告表及总结资料需使用的数据和资料。

（7）总结和分析临床试验结果时，必须采用规范的统计学分析方法，并请熟悉生物统计学的人员参与。

（9）为确保试验数据的可靠性、完整性，临床研究中心的主要研究者、申办者及其委托的CRO监查人员，定期对临床医院进行系统的监查，以判定试验的执行是否与试验方案相符，报告的数据是否与临床参加单位记录一致，每次监查和访视均应写出访视报告。

（10）为了更好地对试验进行监督和监查，研制单位和临床研究负责单位不定期对各临床试验医院进行稽查。以判定试验的实施、数据的记录和分析是否与试验方案、临床试验管理规范和法规的要求相符。稽查结束应写出稽查报告。

## 15.7 资料保存

（1）原始资料由研究单位保管。

（2）申办者认为如有必要，可建立所有或部分受试者的计算机数据库。

（3）申办者和临床研究负责人如有需要，履行一定手续后，有权调阅或查阅所有受试者原始病历。

（4）保存期至试验结束后5年。

（5）本次临床试验的所有资料，所有权属于中山大学附属第一医院，除国家食品药品监督管理局要求外，未经申办者书面同意，研究者不得以任何形式提供给第三者。

## 15.8 项目进度计划

自患者接受治疗之日起，研究的时间暂定为30个月。

# 16. 论文发表

研究者及研究中心其他工作人员对由中山大学附属第一医院提供的所有资料和在研究过程中由参与研究的中心产生的所有数据（受试者的医疗记录除外）应予以保密。研究者或中心的其他工作人员除了用于此研究外不得将资料、数据或记录用于其它目的。这些限制不适用于：（1）不是因研究者或研究中心工作人员的错误致资料已公开发表；（2）出于取信于学术委员会或伦理委员会以评估此研究的目的必须予以公开的资料；（3）为了给参加研究的受试者提供适当的医疗保健必须予以公开的资料；或（4）可按下段文字描述的方法将研究结果予以发表。

研究单位总结、投稿、发表前须经申办单位及临床研究负责单位主要研究者审阅，以事先获得书面同意。

# 17. 参考文献

1. Smith G, Clark J M. Elimination of fluoroscopy use in a pediatric electrophysiology laboratory utilizing three-dimensional mapping.[J]. Pacing Clin Electrophysiol, 2007, 30(4):510-518.
2. Earley M J, Showkathali R, Alzetani M, et al. Radiofrequency ablation of arrhythmias guided by non-fluoroscopic catheter location: a prospective randomized trial. [J]. Eur Heart J, 2006, 27(10):1223-1229.
3. Casella M, Pelargonio G, Dello R A, et al. "Near-zero" fluoroscopic exposure in supraventricular arrhythmia ablation using the EnSite NavX mapping system: personal experience and review of the literature.[J]. J Interv Card Electrophysiol, 2011, 31(2):109-118.
4. Long D Y, Dong J Z, Liu X P, et al. Ablation of right-sided accessory pathways with atrial insertion far from the tricuspid annulus using an electroanatomical mapping system.[J]. J Cardiovasc Electrophysiol, 2011, 22(5):499-505.
5. Committee to Assess Health Risks from Exposure to Low Levels of Ionizing Radiation; Nuclear and Radiation Studies Board, Division on Earth and Life Studies, National Research Council of the National Academies. Health risks from exposure to low levels of ionizing radiation: BEIR VII phase 2. The National Academies Press, Washington, DC, 2006.
6. Alvarez M, Tercedor L, Almansa I, et al. Safety and feasibility of catheter ablation for atrioventricular nodal re-entrant tachycardia without fluoroscopic guidance.[J]. Heart Rhythm, 2009, 6(12):1714-1720.
7. 屈百鸣，钱琳艳，车贤达，等. 三维电场导航系统非透视引导下导管消融治疗阵发性室上性心动过速 [J] .中国心脏起搏与电生理杂志,2011,25(2):108
8. 谭海斌，杨希立，温旭涛，等. Ensite NavXTM三维标测系统指导下零X线曝光消融治疗阵发性心动过速 [J].中国心脏起搏与电生理杂志,2013,27(1):22
9. Sporton S C, Earley M J, Nathan A W, et al. Electroanatomic versus fluoroscopic mapping for catheter ablation procedures: a prospective randomized study.[J]. J Cardiovasc Electrophysiol, 2004,15(3):310-315.
10. 国家食品药品监督管理局. 药物临床试验质量管理规范(GCP2003版).
11. 《药品管理法》、《药品管理法实施条例》及国家食品药品监督管理局有关法规.
12. 新药(西药)临床研究指导原则.卫生部药政局(1993版).

# 附件：临床试验方案确认签字页

| **比较三维标测系统和传统标测系统在右侧旁路射频消融中有效性和安全性的单中心、随机、平行对照临床试验** |
| --- |

**主要研究者关于方案的同意书：**

我已经认真阅读过本方案，我同意方案中包括的所有用来进行研究的必要的信息，并且我同意按方案所描述的内容执行。我明白缺少伦理委员会批准的情况下，试验不得启动，并且要完全遵守本单位的相关规定。

需要获得所有参加试验的受试者的知情同意书和相应的记录文件。签署知情同意之后，将依据赫尔辛基宣言，以及关于射频消融治疗临床应用的法律法规的要求，开展临床试验。

主要研究者姓名：

研究中心编号：

研究中心名称：

研究中心地址：

主要研究者签字： 日期：

**附件： 临床试验方案确认签字页**

| **比较三维标测系统和传统标测系统在右侧旁路射频消融中有效性和安全性的单中心、随机、平行对照临床试验** |
| --- |

**数据管理与统计分析人员关于方案的同意书：**

我已经认真阅读过本方案，我同意方案中包括的所有用来进行研究的必要的信息，并且我同意按方案所描述的内容执行。我明白缺少伦理委员会批准的情况下，试验不得启动，并且要完全遵守本单位的相关规定。

**数据管理与统计分析单位：**

中山大学附属第一医院心血管医学部

签名： 日期：

**附件**：**临床试验方案确认签字页**

| **比较三维标测系统和传统标测系统在右侧旁路射频消融中有效性和安全性的单中心、随机、平行对照临床试验** |
| --- |

**申办者关于方案的同意书：**

我已经认真阅读过本方案，我同意方案中包括的所有用来进行研究的必要的信息，并且我同意按方案所描述的内容执行。我明白缺少伦理委员会批准的情况下，试验不得启动，并且要完全遵守本单位的相关规定。

**申办单位：**

中山大学附属第一医院心血管医学部

签名： 日期：

**附件：临床试验方案确认签字页**

| **比较三维标测系统和传统标测系统在右侧旁路射频消融中有效性和安全性的单中心、随机、平行对照临床试验** |
| --- |

**合同研究组织关于方案的同意书：**

我已经认真阅读过本方案，我同意方案中包括的所有用来进行研究的必要的信息，并且我同意按方案所描述的内容执行。我明白缺少伦理委员会批准的情况下，试验不得启动，并且要完全遵守本单位的相关规定。

**合同研究组织：**

中山大学附属第一医院心血管医学部

签名： 日期：
